# Plasticity in the Glucagon Interactome Reveals Novel Proteins That Regulate Glucagon Secretion in α-TC1-6 Cells

**DOI:** 10.3389/fendo.2018.00792

**Published:** 2019-01-18

**Authors:** Farzad Asadi, Savita Dhanvantari

**Affiliations:** ^1^Department of Pathology and Laboratory Medicine, Schulich School of Medicine and Dentistry, The University of Western Ontario, London, ON, Canada; ^2^Department of Medical Biophysics, Schulich School of Medicine and Dentistry, The University of Western Ontario, London, ON, Canada; ^3^Metabolism, Diabetes and Imaging Programs, Lawson Health Research Institute, London, ON, Canada

**Keywords:** glucagon, alpha cell, proteomics, co-immunoprecipitation, confocal microscopy, glucagon interactome, glucagon secretion

## Abstract

Glucagon is stored within the secretory granules of pancreatic alpha cells until stimuli trigger its release. The alpha cell secretory responses to the stimuli vary widely, possibly due to differences in experimental models or microenvironmental conditions. We hypothesized that the response of the alpha cell to various stimuli could be due to plasticity in the network of proteins that interact with glucagon within alpha cell secretory granules. We used tagged glucagon with Fc to pull out glucagon from the enriched preparation of secretory granules in α-TC1-6 cells. Isolation of secretory granules was validated by immunoisolation with Fc-glucagon and immunoblotting for organelle-specific proteins. Isolated enriched secretory granules were then used for affinity purification with Fc-glucagon followed by liquid chromatography/tandem mass spectrometry to identify secretory granule proteins that interact with glucagon. Proteomic analyses revealed a network of proteins containing glucose regulated protein 78 KDa (GRP78) and histone H4. The interaction between glucagon and the ER stress protein GRP78 and histone H4 was confirmed through co-immunoprecipitation of secretory granule lysates, and colocalization immunofluorescence confocal microscopy. Composition of the protein networks was altered at different glucose levels (25 vs. 5.5 mM) and in response to the paracrine inhibitors of glucagon secretion, GABA and insulin. siRNA-mediated silencing of a subset of these proteins revealed their involvement in glucagon secretion in α-TC1-6 cells. Therefore, our results show a novel and dynamic glucagon interactome within α-TC1-6 cell secretory granules. We suggest that variations in the alpha cell secretory response to stimuli may be governed by plasticity in the glucagon “interactome.”

## Introduction

Glucagon is the major glucose counter-regulatory hormone, and maintains euglycemia by enhancing hepatic gluconeogenesis and glycogenolysis (1). However, both type 1 and type 2 diabetes are characterized by varying levels of hyperglucagonemia (2), which paradoxically exacerbates the hyperglycemia of diabetes (3, 4). More recently, it has been shown that glucagon may be an amino acid regulatory hormone, suggesting a link between hepatic amino acid metabolism and hyperglucagonemia (5). In pancreatic alpha cells, glucagon secretion is tightly regulated by nutritional, hormonal, and neural effectors to maintain normal glucose homeostasis. However, in diabetes, this tight coupling is disrupted (6), resulting in dysfunctional glucagon secretion, which may be a factor in the development of type 2 diabetes (7). This abnormal glucagon secretion has led to strategies (8) to control glucagon action to ameliorate the hyperglycemia of diabetes, such as administering glucagon receptor antagonists or neutralizing antibodies against the glucagon receptor (9, 10). Although effective in the short term, this strategy tends to increase alpha cell mass and worsen alpha cell dysfunction over the long term (6). Therefore, a preferable strategy may be to control the secretion, rather than the action, of glucagon for improved glycemic control in diabetes.

In the context of the pancreatic islet, there is some debate as to whether glucagon secretion is primarily regulated by the paracrine influence of the beta cell, or through intrinsic factors (11, 12). Both insulin and GABA secreted from the beta cell strongly inhibit glucagon secretion, as does somatostatin (13, 14). However, these actions are dependent on prevailing glucose concentrations; at 5 mM glucose, both glucagon and insulin secretion are maximally suppressed (11), suggesting that intrinsic factors may exert an equally prominent influence on glucagon secretion. Some proposed mechanisms of intrinsic regulation of glucagon secretion include glucose metabolic-induced changes in Ca^2+^ and K^+^ membrane conductances or intracellular Ca^2+^ oscillations (15, 16). Intrinsic factors can also include proteins involved in the intracellular trafficking of glucagon. We have previously shown that prolonged culture of α-TC1-6 cells in medium containing 25 mM glucose resulted in the up-regulation of components of the regulated secretory pathway (17), notably proteins associated with secretory granules, such as SNARE exocytotic proteins and granins. There may be direct interactions between granule proteins, such as chromogranin A and carboxypeptidase E, to ensure proper trafficking of glucagon into secretory granules (18), and distinct sorting signals within glucagon may mediate these interactions (19). Therefore, proteins within the alpha cell secretory granules that directly interact with glucagon may provide additional clues for the regulation of glucagon secretion.

In order to identify networks of secretory granule proteins that interact with glucagon, we have continued to use the α-TC1-6 cell line, as this is a well-established cell line in which to study the intrinsic regulation of glucagon secretion (20). This cell line has been extensively used to study glucagon secretory pathway (17, 21) due to its resemblance to the normal pancreatic α-cell in terms of proglucagon processing (22) and response to insulin and somatostatin and nutritional effectors (12, 17). Our work has revealed a novel glucagon “interactome” that exhibits plasticity in response to glucose, insulin and GABA, and contains some novel glucagon-interacting proteins that may regulate glucagon secretion in α-TC1-6 cells.

## Materials and Methods

Sources for all reagents, assays, and software packages are listed in Supplementary Table 1.

### Gene Construct and Plasmid Preparation

We designed a glucagon fusion construct [Fc-glucagon –pcDNA3.1(+)] as follows: the amino acid sequence of glucagon derived from human proglucagon (GenScript, USA; http://www.genscript.com) was fused to the 3′ end of cDNA encoding the CH2/CH3 domain of mouse IgG-2b (Fc), preceded by a 28 amino acid signal peptide as described previously (19). As a negative control for all transfections, proteomics, immunofluorescence microscopy, and co-immunoprecipitation experiments, we also designed a Fc-pcDNA3.1(+) construct. DNA sequences were confirmed at the London Regional Genomics Facility, Western University.

### Extraction and Enrichment of Secretory Granules

Wild type α-TC1-6 cells (a kind gift from C. Bruce Verchere, Vancouver, BC) were cultured in DMEM containing 25 mM glucose, L-glutamine, 15% horse serum, and 2.5% fetal bovine serum, as described previously (17, 23). Based on the ATCC product sheet, the base cell culture medium for α-TC1-6 cells is low glucose (5.5 mM) Dulbecco's Modified Eagle's Medium (DMEM); however, for glucagon secretion (glucagon hypersecretion) studies, high glucose DMEM (16.7 or 25 mM) has been traditionally used to prepare α-TC-6 cells for downstream experiments (17, 24). Cells were grown to 90% confluency and transfected with Fc alone or Fc-glucagon using Lipofectamine 2000. To determine changes in granule size, mass, and proteome, cells were incubated with or without GABA (25 μM), insulin (100 pM), or GABA (25 μM) plus insulin (100 pM) in either 25 or 5.5 mM glucose prior to the granule enrichment procedure. To account for all potential modulators of glucagon secretion, including the possibility of autocrine regulation of glucagon secretion (25, 26) we chose long-term cumulative incubation that has previously been used by our team (17) and other investigators (23) for secretion studies in α-TC1-6 cells.

At the end of the incubation period, granules were extracted as previously published (27) with some modifications. Briefly, cells were detached using 5 mM EDTA in PBS (pH 7.4) containing cOmplete Mini Protease Inhibitor Cocktail (Supplementary Table 1) on ice, centrifuged and resuspended in ice-cold homogenization buffer (20 mM Tris-HCl pH7.4, 0.5 mM EDTA, 0.5 mM EGTA, 250 mM sucrose, 1 mM DTT, cOmplete Mini Protease Inhibitor Cocktail, and 5 μg/mL Aprotinin). The cells were passed 10 times through a 21G needle and again 10 times through a 25G needle. The resulting lysates were centrifuged to obtain a post-nuclear supernatant (PNS). The nuclear fraction was washed seven times in ice-cold homogenization buffer and stored at −80°C. The post-nuclear supernatant (PNS) was centrifuged at 5,400 × g for 15 min at 4°C to obtain a post-mitochondrial supernatant, which then was spun at 25,000 × g for 20 min, and the resultant pellet was washed five times at 4°C. Enrichment was confirmed through immunoblotting for organelle-specific markers as described below.

### Immunoblotting for Organelle-Specific Markers

The enriched preparations of secretory granules from α-TC1-6 cells were lysed using non-ionic lysis buffer (50 mM Tris pH 7.4, 150 mM NaCl, 1% Triton X-100 plus cOmplete Mini Protease Inhibitor Cocktail, and 5 μg/mL Aprotinin). Proteins were resolved by 4–12% NuPAGE, transferred to a PVDF membrane and probed with the following antibodies (Supplementary Table 1): vesicle-associated membrane protein 2 (VAMP2) for mature secretory granules; calreticulin for the endoplasmic reticulum; TGN46 for the trans-Golgi network; and Lamin B1 for the nuclear envelope. Immunoreactive bands were visualized using HRP-conjugated goat anti-rabbit secondary antibody and Clarity Western ECL substrate. Images were acquired on a BioRad ChemiDoc Imaging System. Total cell extracts were used as positive controls.

### Nanoscale Flow Cytometry

#### Secretory Granule Preparation

We used nano-scale flow cytometry (A50-Micro nanoscale flow cytometer; Apogee FlowSystems Inc.) to confirm enrichment of the secretory granules and to determine the size distribution of the granules. α-TC1-6 cells were transfected with Fc-glucagon or Fc alone, and secretory granules were extracted as described above. Granules were fixed in freshly prepared 2% PFA (pH 7.4), permeabilized with 0.5% saponin at room temperature, centrifuged at 25,000 × g for 20 min at 4°C and washed three times in 0.1% saponin in PBS. Fc-containing granules were labeled with FITC-IgG (1:250 dilution in 0.1% saponin in 1% BSA/PBS) in the dark for 1 h, and diluted 200X in 0.1% saponin.

#### Size Calibration

Secretory granules of non-transfected cells were used for size calibration. ApogeeMix beads were used to establish sizing gates along the Y axis—large angle light scattering (LALS) vs. X-axis- small angle light scattering (SALS) plot. The microparticle mixture contained plastic spheres with diameters of 180, 240, 300, 590, 880, and 1,300 nm with refractive indexes of 1.43 and 110 nm, and 500 nm green fluorescent beads with refractive index of 1.59. Based on the manufacturer's default settings, the calibrated gates of the size distribution were 110, 179, 235, 304, 585, and 880 nm, which were used to categorize subpopulations of the enriched secretory granules.

#### Nano-Flow Analysis

To count the numbers of Fc-glucagon^+^ granules, fluorescence of FITC excitation (L488) was gated and the numbers of Fc-glucagon^+^ granules were counted at 110, 179, 235, 304, 585, and 880 nm within the LALS vs. L488 plot. To get the LALS vs. L488 plot, its gate was normalized for the following isotypes: secretory granules of non-transfected cells, secretory granules of Fc-transfected cells, FITC-IgG and diluent. This method resulted in size distributions of the granules that were positive for Fc-glucagon, specifically. All experiments were done in three biological samples and values were expressed as percent distribution of gated granules.

### Proteomic Analysis of Secretory Granule Proteins Associated With Glucagon

#### Granule Lysate Preparation

α-TC1-6 cells were transfected by Fc-glucagon and treated with effectors (GABA, insulin and GABA plus insulin) in media containing 25 or 5.5 mM glucose as described above. To identify non-specific interactors, we used the Fc construct in untreated conditions. Secretory granules were extracted as described above, and lysed in a non-ionic lysis buffer.

#### Affinity Purification

Fc or Fc-glucagon was purified from the granule lysate by immunoprecipitation as we have done previously (19). Briefly, a slurry of Protein A-Sepharose beads (Supplementary Table 1) was mixed 1:1 with the granule lysate and rotated overnight at 4°C. The mixture was then centrifuged at 500 × g for 2 min at 4°C and the pellet was washed twice with 50 mM Tris (pH 7.5) and once with pre-urea wash buffer (50 mM Tris pH 8.5, 1 mM EGTA, 75 mM KCl). Fc or Fc-glucagon was eluted with two volumes of urea elution buffer (7 M urea in 20 mM Tris buffer pH 7.5 plus 100 mM NaCl). This step was repeated twice more and the supernatants were collected and pooled. The pooled supernatant was mixed with acetone in a 1:4 ratio and kept at −20°C overnight, then centrifuged at 16,000 × g for 15 min at 4°C. The pellet was air-dried for proteomic analysis.

#### Proteomic Analysis

Protein identification was conducted using LC-MS/MS according to the protocols of the Western University Mass Spectrometry Laboratory (https://www.schulich.uwo.ca/lrpc/bmsl/protocols/index.html). Briefly, the air-dried pellet was reconstituted in 50 mM NH_4_CO_3_, and proteins were reduced in 200 mM dithiothreitol (DTT), alkylated in freshly prepared 1M iodoacetamide and digested with trypsin for 18 h at 37°C with occasional shaking. Tryptic peptides were acidified using formic acid (0.25; v/v), loaded onto a Hypersep C18 column, washed, and eluted in 50% acetonitrile. The eluent was dried down in a speed vacuum and reconstituted in acetonitrile. Each experimental condition was done in three biological replicates. Peptide sequences were identified using the mouse database and further analyzed for protein categorization through PANTHER GO (www.Pantherdb.org), functional protein-protein interaction clustering through http://string-db.org and determination of subcellular locations and activity using www.uniport.org.

Proteins that were pulled down using Fc alone were subtracted from proteins pulled down by Fc-glucagon to obtain the profile of proteins that specifically interact with glucagon.

### Immunoprecipitation-Immunoblotting of Proteins Associated With Glucagon

To validate the interaction of glucagon with either GRP78 or histone H4 within secretory granules we first purified Fc-glucagon or Fc (as control) from the secretory granule preparation by incubating the secretory granule lysate with Protein A-Sepharose beads overnight at 4°C with rotation. The Fc or Fc-glucagon complex was eluted from the beads with 0.1 M glycine buffer (pH 2.8). The eluate was concentrated 50 times using a speed vac, run on a 10% Bis-Tris NuPAGE gel (Supplementary Table 1) and proteins were transferred onto a PVDF membrane. After an overnight incubation with primary antibodies against GRP78 or histone H4, bands were visualized with HRP-conjugated goat anti-rabbit secondary antibody and Clarity Western ECL substrate (Supplementary Table 1). Images were acquired on a BioRad ChemiDoc Imaging System.

### Histone H4 Assay

Enriched secretory granule fractions were prepared, resuspended in 0.2 N HCl, passed 10 times through a 30G needle, and kept at 4°C overnight. The reaction was stopped by addition of 0.2 volumes of 1N NaOH. The supernatant was collected after centrifugation at 6,500 × g at 4°C for 10 min. Protein levels were determined by BCA assay, and 100 ng of protein was used for measuring total histone H4 (Histone H4 Modification Multiplex ELISA-like format Kit, Supplementary Table 1), as per the manufacturer's instructions. The nuclear fraction was also assayed for histone H4 as a positive control.

### Immunofluorescence Microscopy

To validate the presence of GRP78 and histone H4 in glucagon-positive secretory granules, α-TC1-6 cells were cultured on collagen1-coated coverslips (three per experiment), and processed for immunofluorescence microscopy as described previously (18). Briefly, cells were fixed in 4% paraformaldehyde and permeabilized in 0.1% saponin in 0.5% BSA for 1 h. After blocking in 10% goat serum, cells were incubated with primary antibodies (mouse anti-glucagon and rabbit anti-GRP78 or rabbit anti-histone H4) overnight. Coverslips were washed in PBS and incubated with goat anti-mouse Alexa Fluor IgG 488 and goat anti-rabbit Alexa Fluor 594 (Supplementary Table 1) for 3 h in the dark at room temperature, then mounted using ProLong Gold Antifade Mountant. Images were acquired on a Nikon A1R Confocal microscope with a 60x Nikon Plan-Apochromat oil differential interference contrast objective lens using NIS-Elements, software. To show secretory granule co-localization, images were post-processed by 2D deconvolution. To measure the degree of co-localization, regions of interest were manually drawn around distinct single or multicell bodies, positive for Fc-glucagon and either GRP78 or histone H4 and cropped for analysis. Co-localization of the pixels from each pseudo-colored image were used to calculate Pearson's correlation coefficient (PCC), as we described previously (19).

### siRNA-Mediated Depletion of Targeted Proteins

After treatment of α-TC1-6 cells with GABA and/or insulin in media containing 25 mM glucose as described above, the proteomes were tabulated, and Venn diagram analysis revealed 27 metabolic/regulatory/secretory proteins and 36 histone/cytoskeletal/ribosomal proteins that were common between the groups treated with GABA and insulin. We selected 11 of these proteins (based on availability of the pre-designed siRNA) for siRNA-mediated depletion: Peroxiredoxin-2 (PRDX2), Malate dehydrogenase 1 (MDH1), Aconitate hydratase, mitochondrial (ACO2), 14-3-3 protein zeta/delta (KCIP-1), ELKS/Rab6-interacting/CAST family member 1 (ERC1), Alpha-tubulin 2 (AT2), ATP synthase F1 subunit alpha (ATP5F1A), Histone H4, GRP78, FXYD domain-containing ion transport regulator 2 (FXYD2), and Protein disulfide-isomerase (PDI), (Silencer siRNA, Thermo Fisher Scientific Inc. MA, USA).

Gene silencing was based on a published protocol (28). Briefly, α-TC1-6 cells were cultured to 60% confluency and transfected with final concentrations of 50 nM of pooled siRNAs (three siRNAs for each target) or control scrambled siRNA using Lipofectamine2000. Cells were incubated for 48 h, after which media were removed and replaced. After 24 h, expression levels of the targeted proteins were evaluated by immunoblotting using primary antibodies against each protein (Supplementary Table 1). Meanwhile, siRNA mediated knockdown of the proglucagon gene was shown as a positive control using real-time PCR (Quant Studio Design and Analysis Real-Time PCR Detection System) (Supplementary Figure 4).

### Glucagon Measurement

To measure cellular and secreted glucagon levels after siRNA-mediated gene silencing, cell lysates or media were acidified in HCl-ethanol (92:2 v/v) in a 1:3 ratio, kept at −20°C overnight, then centrifuged at 13,000 × g for 15 min at 4°C. The supernatant was then mixed 1:1 with 20 mM Tris, pH 7.5 26 and glucagon levels were measured by ELISA (Thermo Fisher Scientific, Supplementary Table 1) according to the manufacturer's instructions. To measure Fc-glucagon, samples were diluted to reach an OD at the linear part of the standard curve.

### Glucagon Secretion and Cell Glucagon Content in Response to Nutritional and Paracrine Effectors

α-TC1-6 cells cultured and kept under chronic exposure to 25 mM glucose and at confluency rate of ~70% were plated out into six-well plates. After 24 h, two sets of experiments were designed. In one set, medium was replaced by fresh 25 mM glucose-containing medium and in the other set medium was replaced by fresh medium containing 5.5 mM glucose. In both sets, cells were treated by GABA (25 μM), insulin (100 pM), or GABA (25 μM) + insulin (100 pM), and incubated for 24 h. At the end of incubation, plates were placed on ice and media were collected, centrifuged at 16,000 × g for 5 min, and supernatant was removed for glucagon measurement. The cells were washed three times with ice-cold PBS and scraped in Glycine-BSA buffer (100 mM glycine, 0.25% BSA, cOmplete Mini Protease Inhibitor Cocktail, 5 μg/mL Aprotinin, pH 8.8). The scraped cells were lysed by sonication (12 s at 30% amplitude on ice), and centrifuged at 16,000 × g for 45 min, from which the supernatant was collected for analysis. The protein concentration of the cell lysate was measured using BCA assay. To measure glucagon levels, the cell lysate or medium was mixed in an ethanol-acid solution (96% ethanol containing 0.18 M HCl) in a 1:3 ratio, kept at −20°C overnight, then centrifuged at 16,000 × g for 15 min at 4°C. The supernatant was then mixed with 20 mM Tris buffer, pH 7.5, and glucagon measurements were conducted by ELISA.

### Statistical Analysis

Experiments were done in three biological replicates, each of which had two technical replicates. Values were compared among treatment groups by one-way ANOVA using Sigma Stat 3.5 software (α = 0.05). For image analysis, co-localization of channels in the merged images was calculated by PCC using NIS-Elements software (Nikon, Canada).

## Results

Our method for purification of proteins that associate with glucagon within the α cell secretory granules consisted of two sequential steps. First, we modified and used a previously published method (27) for enrichment of the secretory granule fraction. Second, we used Fc-glucagon for affinity purification to pull down proteins associated with glucagon within the secretory granules.

### Secretory Granule Enrichment

Immunoblotting for organelle-specific markers confirmed enrichment of secretory granules (Supplementary Figures 1A–D). The final granule fraction was positive for the secretory granule marker, VAMP2. In contrast, the granule fraction did not contain the trans-Golgi marker TGN46, the nuclear envelope marker LaminB1, or the endoplasmic reticulum marker Calreticulin. As a positive control, the general cell lysate contained all four markers.

### Confirmation of the Enriched Secretory Granules

Secretory granules in alpha cells have been previously studied using transmission electron microscopy and their average sizes have been reported to be in the range of 180–240 nm (29–31). Accordingly, we confirmed the presence of secretory granules using nano-scale flow cytometry with Fc-glucagon as an exclusive marker for alpha cell secretory granules (32, 33). We used beads in the range of 110–880 nm for calibration in the range of the reported sizes for secretory granules (Supplementary Figure 2A). Fc-glucagon^+^ secretory granules distributed mostly to the gated regions of 179 and 235 nm (Supplementary Figures 2B,C), confirming enrichment of secretory granules from α-TC1-6 cells.

### Proteomic Analysis of Proteins That Are Associated With Glucagon Within Alpha Cell Secretory Granules

**Figure 1:**
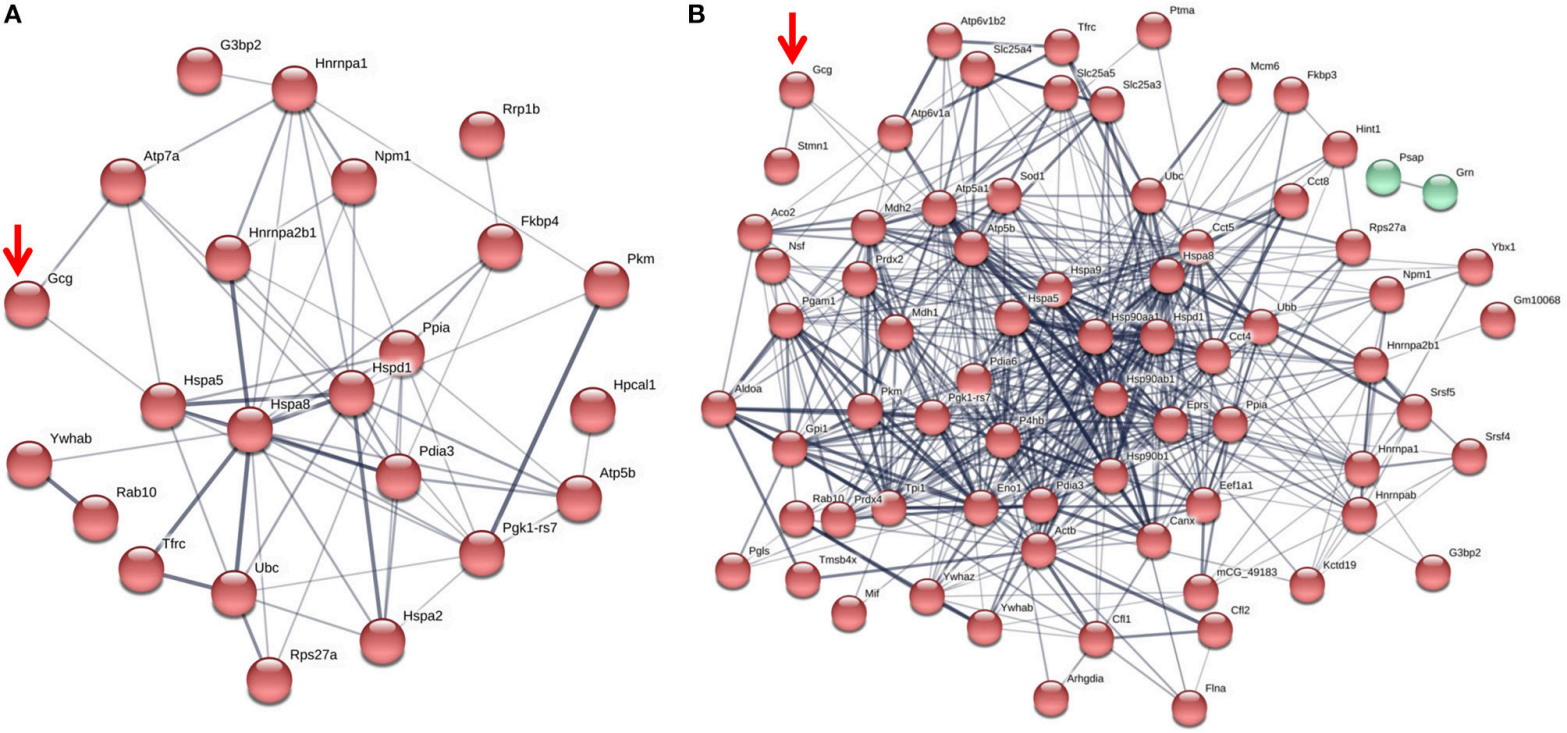
The glucagon interactome in secretory granules of α-TC1-6 cells. Cells were transfected with Fc-glucagon or Fc alone, and cultured in DMEM containing 25 or 5.5 mM glucose for 24 h. Fc-glucagon was purified from enriched secretory granules and associated proteins were identified by LC-MS/MS. **(A)** Proteomic map of the metabolic-regulatory-secretory proteins that are predicted to associate with glucagon in the context of 25 mM glucose. Network clustering predicts direct interactions between glucagon and glucose regulated protein 78 KDa (Hspa5, also known as Grp78), and ATPase copper transporting alpha polypeptide (Atp7). **(B)** Proteomic map of the metabolic-regulatory-secretory proteins that are predicted to associate with glucagon in the context of 5.5 mM glucose. Network clustering predicts direct interactions between glucagon and GRP78, stathmin1 (Stmn1), and heat shock protein 90-alpha (Hsp90aa1). The thickness of the lines indicate the strength of the predicted protein-protein interaction.

Fc or Fc-glucagon was purified from the granule lysate by affinity purification, and proteins that interact with either Fc alone or Fc-glucagon were identified with LC-MS/MS. Proteins that were pulled down by Fc alone in both 25 mM glucose (Supplementary Table 2) and 5.5 mM glucose (Supplementary Table 3) conditions were subtracted from the list of proteins identified using Fc-glucagon, thus identifying proteins that specifically interact with glucagon, which we term the glucagon interactome. Proteins were assigned the following categories: metabolic-secretory-regulatory, histones, cytoskeletal, and ribosomal. We identified 42 and 96 glucagon-interacting proteins within the category of metabolic-regulatory-secretory proteins when the cells were cultured in media containing 25 mM (Figure 1A) and 5.5 mM glucose (Figure 1B), respectively.

In media containing 25 mM glucose, there was a predicted direct interaction of glucagon with glucose regulated protein 78 kDa (GRP78 or Hspa5), and ATPase copper transporting alpha polypeptide (Atp7a) (Figure 1A), while in media containing 5.5 mM glucose, GRP78, Stathmin1 (Stmn1), and Heat shock protein 90- alpha (Hsp90aa1) were predicted to directly interact with glucagon (Figure 1B). Under conditions of either 25 or 5.5 mM glucose, one common predicted interaction was that between glucagon and GRP78.

### GRP78 Interacts With Glucagon and Co-localizes to Glucagon-Positive Secretory Granules

**Figure 2:**
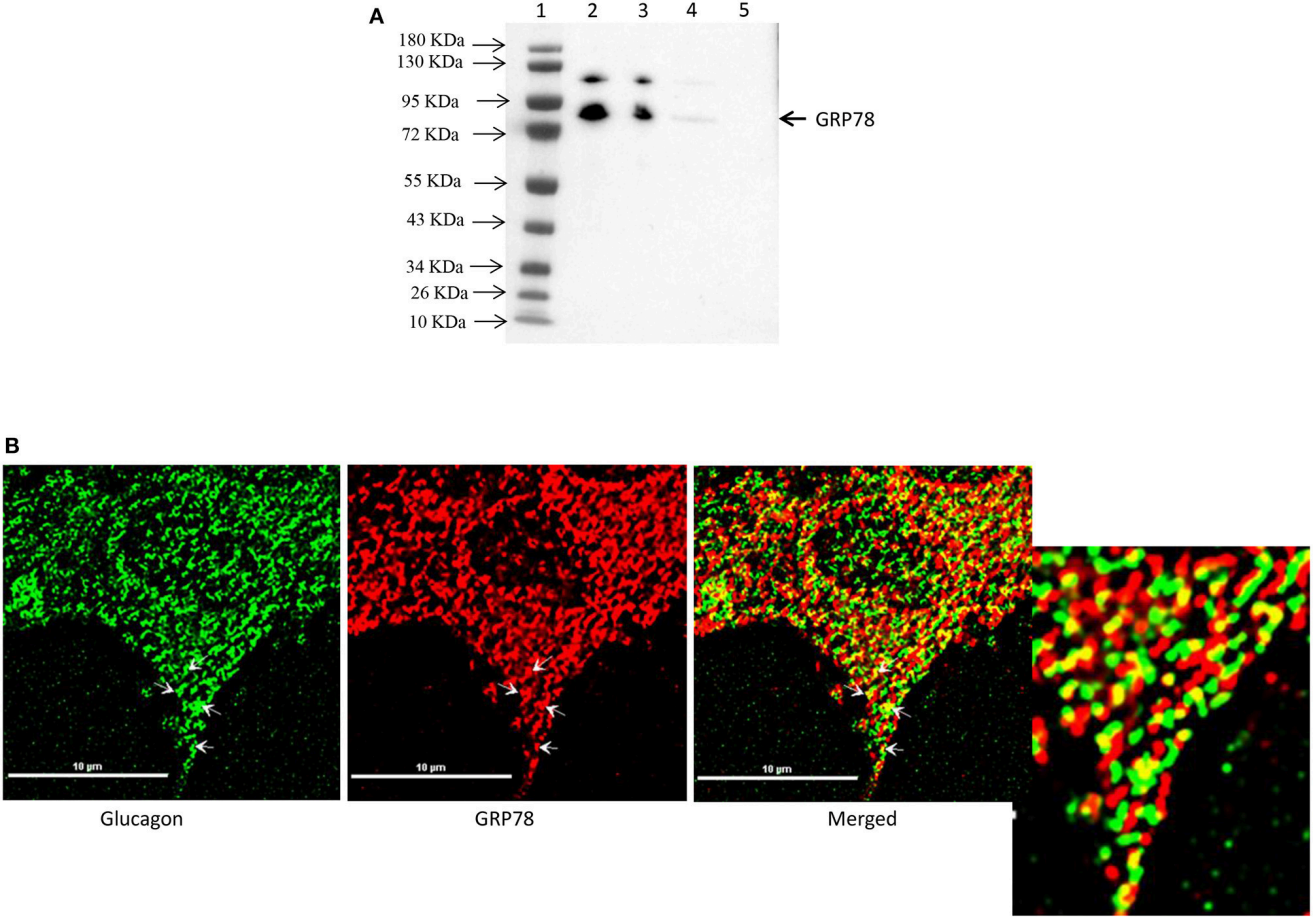
Glucagon and GRP78 directly interact and are localized within secretory granules in α-TC1-6 cells. **(A)** Western blot showing GRP78 immunoreactivity in: total cell extracts from untransfected (lane 2) and transfected (lane 3) cells; affinity-purified Fc-glucagon from isolated secretory granules (lane 4); and affinity-purified Fc alone from isolated secretory granules (lane 5). GRP78 binds to Fc-glucagon, but not Fc alone. **(B)** Immunofluorescence microscopy of glucagon (green), GRP78 (red) and both images merged. Cells were cultured on collagen-coated coverslips for 24 h in DMEM containing 25 mM glucose. Images were acquired, 2D deconvoluted and analyzed with NIS-Elements, software (Nikon, Canada). Pearson correlation coefficient (PCC) indicates strong correlation between GRP78 and glucagon (PCC = 0.85 ± 0.08). ROI shows areas of colocalization of GRP78 and glucagon within secretory granules.

Affinity purification of Fc-glucagon or Fc alone from the secretory granule lysate was followed by immunoblotting for GRP78. The presence of GRP78 immunoreactivity with Fc-glucagon, and not Fc alone, demonstrates a direct interaction with glucagon in the enriched secretory granules (Figure 2A). Immunofluorescence microscopy showed co-localization of GRP78 and endogenous glucagon within the secretory granules in α-TC1-6 cells (Figure 2B). There was a strong positive correlation between glucagon and GRP78 immunoreactivities (PCC = 0.85 ± 0.08), indicating significant co-localization of GRP78 and glucagon.

### GABA Induces Histone H4 Interaction and Co-localization With Glucagon

**Figure 3:**
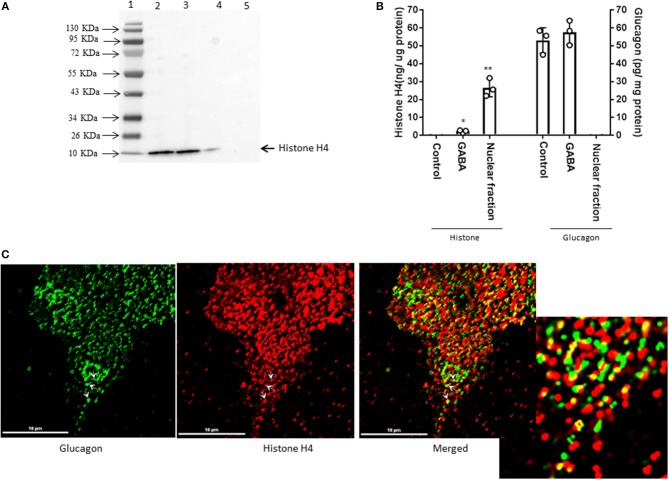
GABA induces direct interaction between glucagon and histone H4 within secretory granules in α-TC1-6 cells. **(A)** Western blot shows histone H4 immunoreactivity in: total cell extracts from untransfected (lane 2) and transfected (lane 3) cells; affinity-purified Fc-glucagon from isolated secretory granules (lane 4); and affinity-purified Fc alone from isolated secretory granules (lane 5). Histone H4 binds to Fc-glucagon, but not Fc alone. **(B)** Quantitative ELISA measurement of histone H4 (left Y axis) and glucagon (right Y axis) within the secretory granules (control GABA, insulin) and the nuclear fraction of α-TC1-6 cells. Values are expressed as mean±SD and compared with 1-way ANOVA (α = 0.05). ^*^p < 0.05; ^**^p < 0.001. **(C)** Immunofluorescence microscopy of glucagon (green), histone H4 (red), and both images merged. Cells were cultured on collagen-coated coverslips for 24 h in DMEM containing 25 mM glucose. Images were acquired, 2D deconvoluted and analyzed with NIS-Elements, software (Nikon, Canada). Pearson correlation coefficient (PCC) indicates strong correlation between histone H4 and glucagon (PCC = 0.78 ± 0.08). ROI shows areas of colocalization of histone H4 and glucagon within secretory granules.

Interestingly, proteomic analysis also revealed the presence of histone proteins, along with structural proteins and ribosomal proteins, within the secretory granules in α-TC1-6 cells (Supplementary Table 4). Histone H4 was predicted to interact with glucagon in cells incubated in medium containing 5.5 mM glucose. Therefore, we reasoned that this interaction was responsive to external effectors. We treated α-TC1-6 cells with GABA, a well-known modulator of glucagon secretion (21) and examined the interaction between histone and glucagon. Co-immunoprecipitation of granule lysates, histone H4 ELISA of granule lysates, and immunofluorescence microscopy all validated the interaction of histone H4 with glucagon and presence of histone H4 in secretory granules of α-TC1-6 cells after treatment with GABA (Figure 3). Affinity purification of Fc-glucagon or Fc alone from the secretory granule lysate was followed by immunoblotting for histone H4 (Figure 3A). The presence of histone H4 immunoreactivity with Fc-glucagon, and not Fc alone, demonstrates a direct interaction with glucagon in the enriched secretory granules (Figure 3A). We then confirmed the presence of histone H4 in the enriched secretory granules of α-TC1-6 cells by ELISA (Figure 3B). In cells treated with GABA in 25 mM glucose, there was a detectable amount of histone H4 in the granules. That this result was not due to contamination from the nuclear fraction was shown by the finding that histone H4 levels were undetectable in the secretory granules of cells not treated with GABA. As a positive control, the nuclear fraction showed high levels of histone H4. Finally, immunofluorescence microscopy showed the presence of histone H4 in glucagon-containing secretory granules (Figure 3C), and there was significant co-localization with glucagon as assessed by Pearson's correlation coefficient (PCC = 0.78 ± 0.08).

### The Glucagon Interactome Changes in Response to Glucose, GABA and Insulin

**Figure 4:**
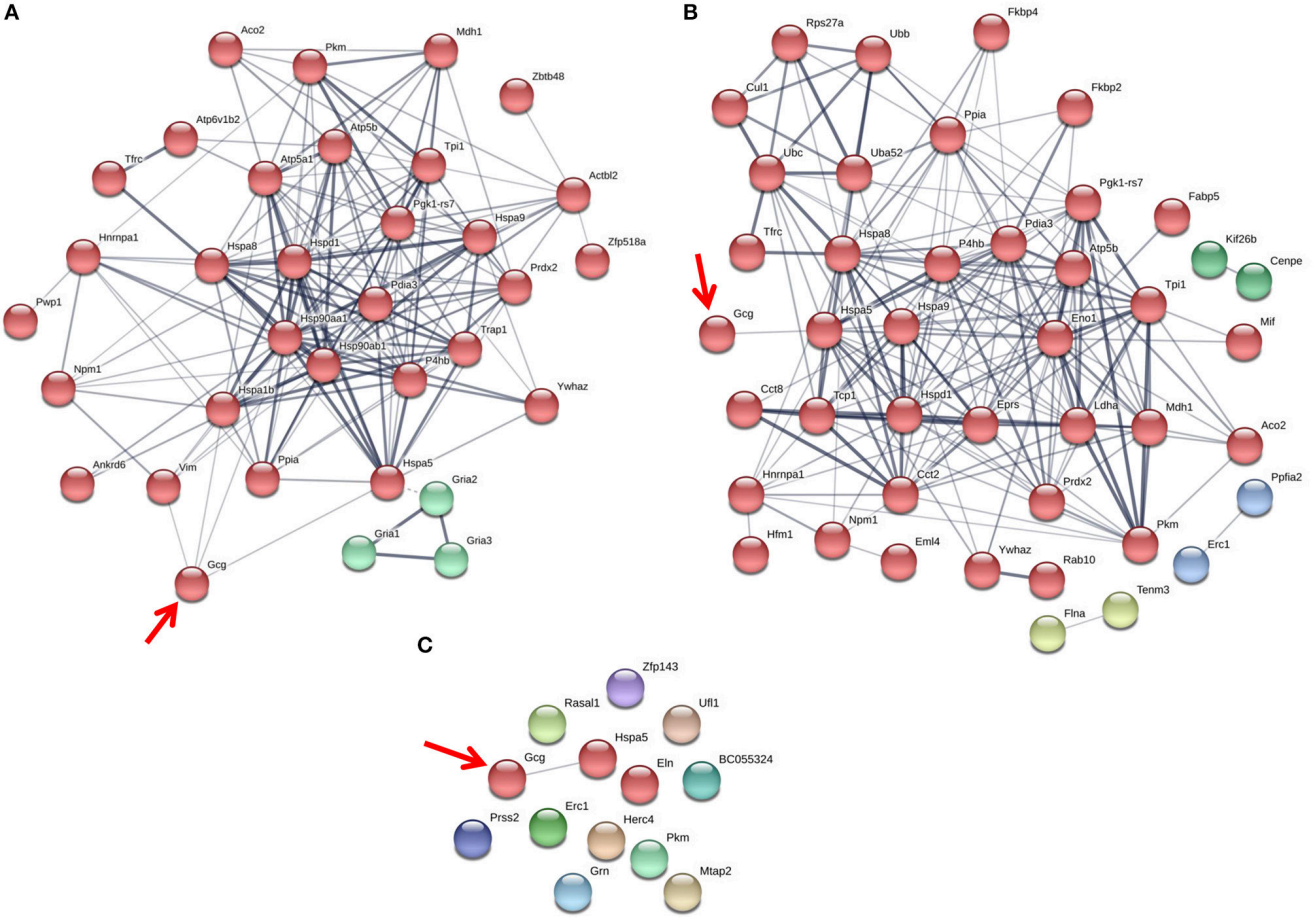
The glucagon interactome is altered in response to paracrine effectors in 25 mM glucose. α-TC1-6 cells were transfected with Fc-glucagon or Fc alone, and treated with GABA (25 μM), insulin (100 pM) or GABA (25 μM) plus insulin (100 pM) for 24 h in DMEM containing 25 mM glucose. Fc-glucagon was purified from isolated secretory granules and associated proteins were identified by LC-MS/MS. **(A)** Proteomic map of metabolic-regulatory-secretory proteins that are associated with glucagon after treatment of α-TC1-6 cells with GABA shows direct interactions with 4 proteins: GRP78, Heat shock 70 kDa protein 1B (Hspa1b) Heat shock protein 90- alpha (Hsp90aa1), and Vimentin (Vim). **(B)** After treatment with insulin or **(C)** GABA + Insulin, glucagon is predicted to interact only with GRP78. Line thickness indicates the strength of data support.

**Figure 5:**
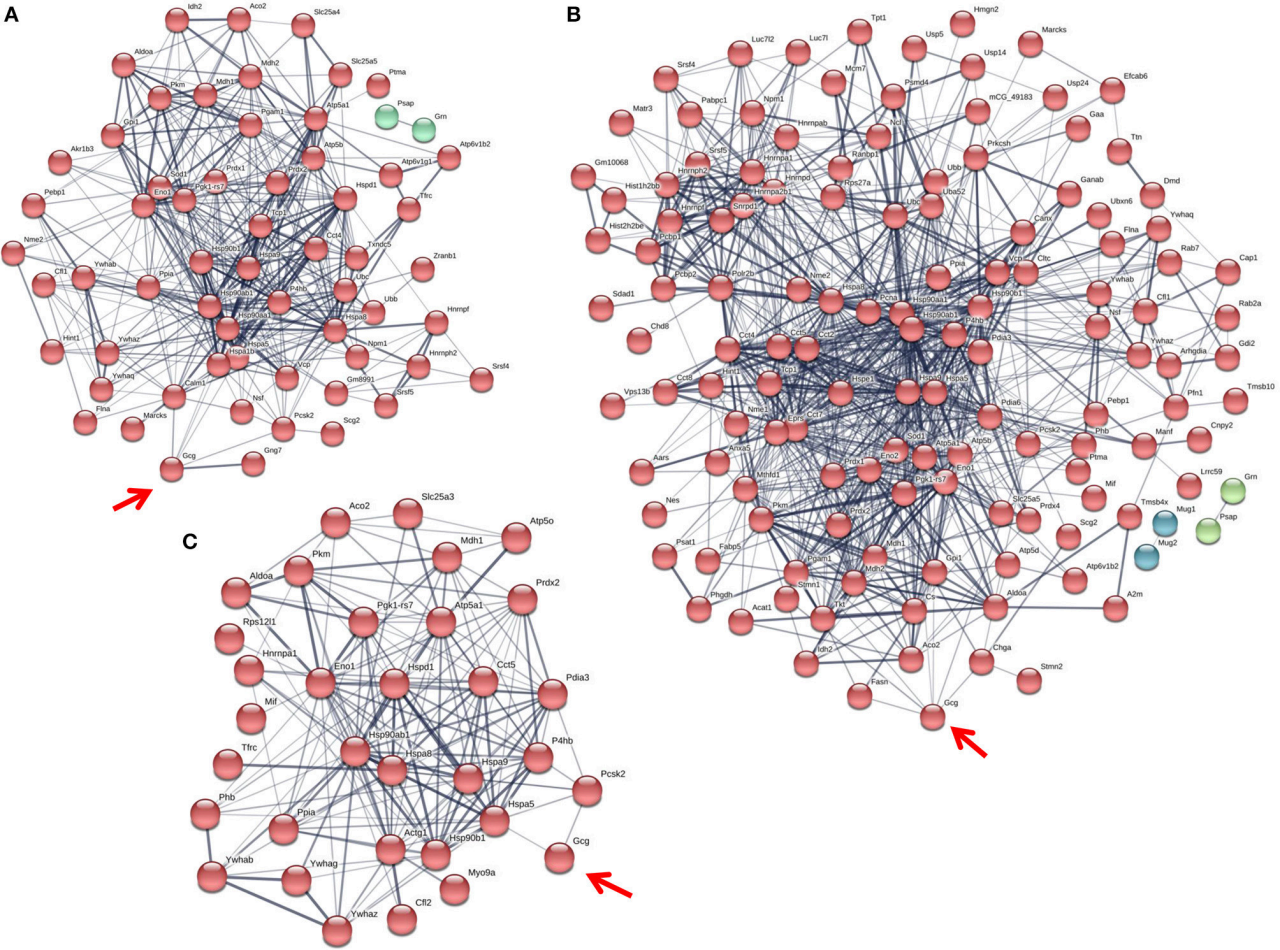
The glucagon interactome is altered in response to paracrine effectors in 5.5 mM glucose. α-TC1-6 cells were transfected with Fc-glucagon or Fc alone, and treated with GABA (25 μM), insulin (100 pM) or GABA (25 μM) plus insulin (100 pM) for 24 h in DMEM containing 5.5 mM glucose. Fc-glucagon was purified from isolated secretory granules and associated proteins were identified by LC-MS/MS. **(A)** Proteomic map of metabolic-regulatory-secretory proteins that are associated with glucagon after treatment of α-TC1-6 cells with GABA shows direct interactions with 6 proteins: GRP78, Heat shock protein 90- alpha (Hsp90aa1), Protein convertase subtilisin/kexin type2 (PCSK2), Heat shock 70 kDa protein 1B (Hspa1b), Calmodulin 1 (Calm1), Guanine nucleotide-binding protein G(I)/G(S)/G(O) subunit gamma-7 (Gng7). **(B)** After treatment with insulin, glucagon is predicted to directly interact with 7 proteins: GRP78, Heat shock protein 90-alpha, Annexin A5 (Anxa5), Stathmin1 (Stmn1), PCSK2, Fatty acid synthase (Fasn), and Chromogranin A (Chga). **(C)** After treatment with GABA + Insulin, glucagon is predicted to directly interact with GRP78 and PCSK2. Line thickness indicates the strength of data support.

Since the interaction between histone H4 and glucagon was dependent on glucose levels and GABA, we determined the effects of the major alpha cell paracrine effectors, GABA and insulin, on the glucagon interactome. The profiles of the metabolic-regulatory-secretory proteins that associate with glucagon within secretory granules were altered upon treatment with GABA, insulin or GABA + insulin, respectively, when α-TC1-6 cells were cultured in medium containing 25 mM glucose (Figure 4) and in 5.5 mM glucose (Figure 5). Additionally, we tabulated the profiles of histone, cytoskeletal, and ribosomal proteins in response to GABA, insulin and GABA + insulin in 25 mM glucose (Supplementary Tables 5A–C) or 5.5 mM glucose (Supplementary Tables 6A–C). The glucagon interactomes were functionally classified into the following groups: Binding, Structural molecule, Catalytic, Receptor, Translation regulator, Transporter, Signal transducer, Antioxidant. The proportion of proteins in each category is shown in the context of 25 mM glucose (Supplementary Table 7) and 5.5 mM glucose (Supplementary Table 8).

The protein networks that are predicted to interact with glucagon within the secretory granules under conditions of 25 mM glucose are illustrated in Figure 4. In cells treated with GABA, glucagon is predicted to directly interact with GRP78, HSP1B, HSP90, and vimentin (Figure 4A); however, in cells treated with insulin and GABA + insulin, glucagon interacts directly with only GRP78 (Figures 4B,C). The clusters of metabolic-secretory-regulatory proteins that make up the rest of the glucagon interactomes change in composition in response to the different treatments. The numbers of proteins categorized as “structural molecule activities” decreased in response to GABA (~45%) or insulin (~38%) and increased in the GABA + insulin group (~16%) compared to the control (Supplementary Table 7). The numbers of cytoskeletal proteins increased in the GABA (29%), insulin (12%), and GABA + insulin (35%) groups, while the numbers of ribosomal proteins decreased in those groups by 51, 14, and 66%, respectively (Table 1A).

**Table 1A:** Sub-groups of proteins categorized as “structural molecules” in the glucagon interactome under conditions of 25 mM glucose.

	**Structural constituent of cytoskeleton**	**Structural constituent of ribosome**	**Extracellular matrix structural constituent**
Control	66.7	29.2	4.2
GABA	85.7	14.3	–
Insulin	75	25	–
GABA + Insulin	90	10	–

*Panther GO-Slim Molecular Function analysis resulted in 3 sub-categories. The values represent protein hits as a percentage of the total number of hits within each sub-category when α-TC1-6 cells were cultured in media containing 25 mM glucose*.

Compared to cells incubated in medium containing 25 mM glucose, there were dramatic increases in the numbers of metabolic-regulatory-secretory proteins associated with glucagon after treatment with GABA, insulin or GABA + insulin in cells incubated in media containing 5.5 mM glucose (Figure 5). In cells treated with GABA, glucagon is predicted to directly interact with the following proteins: GRP78 (Hspa5), HSP 90alpha (Hsp90aa1), proprotein convertase subtilisin/kexin type 2 (PCSK2), heat shock 70 kDa protein 1B (Hsp1b), calmodulin 1(Calm1), and guanine nucleotide-binding protein G(I)/G(S)/G(O) subunit gamma-7 (Gng7) (Figure 5A). Under insulin treatment, the following proteins were predicted to directly interact with glucagon: GRP78, HSP 90-alpha, annexin A5 (Anxa5), stathmin1 (Stmn1), fatty acid synthase (Fasn), and chromogranin A (Chga) (Figure 5B); and only two proteins, GRP78 and PCSK2, were predicted to directly interact with glucagon after treatment with GABA + insulin (Figure 5C).

In the context of 5.5 mM glucose, the number of cytoskeletal proteins decreased, and the number of ribosomal proteins increased compared to cells treated with GABA, insulin and GABA + insulin in 25 mM glucose (Table 1B). Interestingly, the total numbers of proteins classified as “structural molecule activities” did not change appreciably across treatments (Supplementary Table 8). However, differences became apparent when cytoskeletal and ribosomal proteins were compared separately. When compared to 5.5 mM glucose alone, there were decreases of ~24 and ~35%, respectively, in the numbers of cytoskeletal proteins when cells were treated with GABA or insulin alone, but a ~71% increase in response to GABA + Insulin. Conversely, the numbers of ribosomal proteins increased by ~26 and ~43% in response to GABA and insulin, respectively, and decreased by ~69% in response to GABA + Insulin (Table 1B).

**Table 1B:** Sub-groups of proteins categorized as “structural molecules” in the glucagon interactome under conditions of 5.5 mM glucose.

	**Structural constituent of cytoskeleton**	**Structural constituent of ribosome**	**Extracellular matrix structural constituent**
Control	50	45.5	4.5
GABA	38.1	57.1	4.8
Insulin	32.4	64.9	2.7
GABA + Insulin	85.7	14.3	–

*Panther GO-Slim Molecular Function analysis resulted in 3 sub-categories. The values represent protein hits as a percentage of the total number of hits within each sub-category when α-TC1-6 cells were cultured in media containing 5.5 mM glucose*.

### The Dynamic Glucagon Interactome Reveals Novel Proteins That Regulate Glucagon Secretion

**Figure 6:**
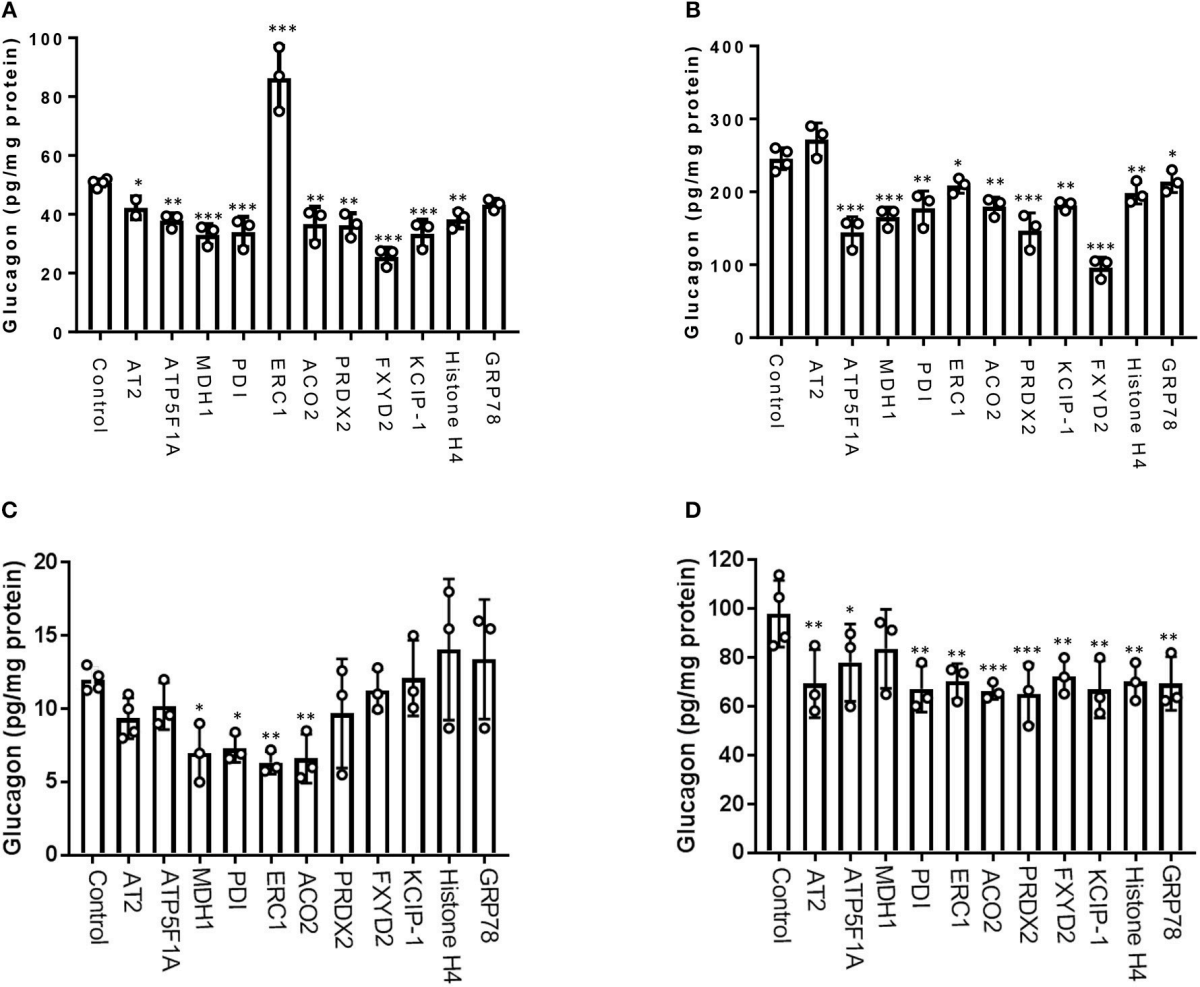
Glucagon secretion and cell content are regulated by a subset of interactome proteins. **(A)** Glucagon secretion and **(B)** cell content in the context of 25 mM glucose, and **(C)** glucagon secretion and **(D)** cell content in the context of 5.5 mM glucose were assessed after siRNA-mediated gene silencing of the following proteins: Alpha-tubulin 2 (AT2), ATP synthase F1 subunit alpha (ATP5F1A), Malate dehydrogenase 1 (MDH1), Protein disulfide-isomerase (PDI), ELKS/Rab6-interacting/CAST family member 1 (ERC1), Aconitate hydratase mitochondrial (ACO2), Peroxiredoxin-2 (PRDX2), 14-3-3 protein zeta/delta (KCIP-1), FXYD domain-containing ion transport regulator 2 (FXYD2), histone H4, and GRP78 using pre-designed siRNAs for the mouse genome. After siRNA transfection, α-TC1-6 cells were cultured in DMEM containing 25 or 5.5 mM glucose for 24 h and glucagon levels were measured using ELISA. Values are expressed as mean ± SD (α = 0.05; *n* = 3–4). ^*^p < 0.05; ^**^p < 0.01; ^***^p < 0.001.

From our glucagon interactomes, we identified 11 proteins that interact with glucagon after treatment of α-TC1-6 cells with either GABA or insulin in media containing 25 mM glucose. To determine their effects on glucagon secretion, these proteins were depleted with siRNAs (Supplementary Figure 3) and glucagon secretion and cell content were measured. Of these 11 proteins, knockdown of ELKS/Rab6-interacting/CAST family member 1 (ERC1) increased glucagon secretion (*p* < 0.001), while gene silencing of 14-3-3 zeta/delta (KCIP-1), cytosolic malate dehydrogenase (MDH1), FXYD domain-containing ion transport regulator 2 (FXYD2) and protein disulfide-isomerase (PDI) reduced glucagon secretion to the same statistically significant level (*p* < 0.001). As well, knockdown of peroxiredoxin-2 (PRDX2), ATP synthase F1 subunit alpha (ATP5F1A), histone H4, and aconitate hydratase mitochondrial (ACO2) reduced glucagon secretion (*p* < 0.01), as did knockdown of alpha-tubulin 2 (AT2) (*p* < 0.05) (Figure 6A). Gene silencing of MDH1, PRDX2, ATP5F1A, and FXYD2 reduced cellular glucagon content to a significance level of *p* < 0.001. Gene silencing of KCIP-1, ACO2, Histone H4 and PDI all reduced the levels of cellular glucagon content to a significance level of *p* < 0.01 and that for ERC1 at *p* < 0.05 (Figure 6B). Gene silencing of GRP78 had no effect on glucagon secretion, and reduced cellular glucagon content (*p* < 0.05).

In the context of 5.5 mM glucose, significant reduction of glucagon secretion occurred by depletion of MDH1 (*p* < 0.05), PDI(*p* < 0.05), ERC1(*p* < 0.01), and ACO2 (*p* < 0.01). However, silencing of the other abovementioned genes did not significantly alter glucagon secretion (Figure 6C). Cellular glucagon content was significantly decreased by silencing of ATP5F1A (*p* < 0.05), AT2, PDI, ERC1, FXYD2, KCIP-1, histone H4, GRP78 (*p* < 0.01), ACO2, and PRDX2 (*p* < 0.001) (Figure 6D).

### Alterations in Glucagon Secretion and Cell Glucagon Content in Response to Nutritional and Paracrine Effectors

α-TC1-6 cells were cultured under high glucose conditions (25 mM) and then treated with paracrine effectors (GABA, insulin or GABA + insulin). The profiles of cumulative glucagon secretion and cellular glucagon content in 25 mM glucose was different from that in 5.5 mM glucose. While neither GABA nor insulin affected glucagon secretion in 5.5 mM glucose, they suppressed glucagon secretion in 25 mM glucose (Supplementary Figure 5A). In the context of 25 mM glucose, GABA reduced cellular glucagon content, while insulin increased cellular glucagon content (Supplementary Figure 5B); in contrast, neither GABA nor insulin alone affected cellular glucagon content, but in combination, they decreased cellular glucagon content.

## Discussion

We have identified a dynamic “glucagon interactome” within secretory granules of alpha cells that is altered in response to glucose levels and the paracrine effectors GABA and insulin. We used a tagged glucagon construct, Fc-glucagon, to bring down proteins within secretory granules. We validated enrichment of the secretory granules by nano-scale flow cytometry and immunoblotting with compartment-specific markers. We identified a network of 392 proteins within the secretory granules that interact with glucagon and showed a direct interaction with GRP78 and Histone H4. Components of the interactome played a role in glucagon secretion, thus revealing a role for the interactome in the regulation of glucagon secretion in α-TC1-6 cells.

We have previously shown that α-TC1-6 cells have elevated levels of both proglucagon mRNA and glucagon secretion in response to 25 mM glucose (17), and other groups have shown the same effect in isolated mouse islets (3), clonal hamster InR1G9 glucagon-releasing cells (3, 34), and perfused rat pancreas (35). We also showed that this paradoxical glucagon release is accompanied by an up-regulation of components of the regulated secretory pathway, particularly in the active forms of PC1/3 and PC2 that post-translationally process proglucagon to glucagon, and in SNARE proteins that mediate vesicle exocytosis (17). Under conditions of 5.5 mM glucose, the up-regulation in RNA-binding proteins that modulate biosynthesis of islet secretory granule proteins, along with chaperonins, may indicate an increase in protein synthesis (36, 37). Chaperonins, as key components of the cellular chaperone machinery, are involved in maturation of newly-synthesized proteins in an ATP dependent manner (36). As ATP-generating proteins, such as ATP5F1A, MDH1, and glucose metabolic proteins, were also increased, we speculate that 5.5 mM glucose induced a stress response that resulted in increased protein translation. This hypothesis is strengthened by the identification of cold shock protein, peroxiredoxin, thiol-disulfide isomerase and thioredoxin within the glucagon interactome at 5.5 mM glucose, all of which are up-regulated in pancreatic islets in response to stress (37).

One protein that was consistently predicted as interacting directly with glucagon was the ER stress protein and molecular chaperone GRP78. Previous proteomic studies have identified GRP78 in islets and beta cells (38, 39). Its presence in alpha cell secretory granules may not be surprising, as it has previously been found in non-ER compartments such as the nucleus and lysosomes. Our data suggest that GRP78 may be a novel sorting receptor for glucagon in the regulated secretory pathway of alpha cells. We have previously shown a potential role of chromogranin A as a sorting receptor for glucagon in both α-TC1-6 cells and PC12 cells (19), but unlike GRP78, we did not demonstrate any direct interactions with glucagon. While knockdown of GRP78 did not reduce glucagon secretion, it did reduce cell content, indicating a potential role in intracellular trafficking, but not exocytosis, of glucagon.

Interestingly, we identified histone proteins as a functional part of the glucagon interactome. The discovery of histone proteins within alpha cell secretory granules is novel, and supported by the findings that the cytosolic fraction of pooled islets from multiple human donors had abundant amounts of the histone H2A (40). As well, quantitative proteomics of both αTC1 and ßTC3 cells revealed the presence of histones H4, H3, H2A, H2B, and H1 (41). Our data indicate that one of these histones, H4, may directly bind to glucagon and regulate its basal level of secretion, perhaps under conditions of stress. Oxidative stress contributes to the pathogenesis of diabetes by disrupting the balance between reactive oxygen species and antioxidant proteins (42). Such an imbalance could target chromatin and globally alter profiles of gene expression, especially those encoding histone and DNA-binding proteins (42, 43). Thus, we speculate that the presence of histone H4 in the secretory granules could reflect a response to microenvironmental stress. Furthermore, it has been suggested that histones contained within secretory granules in neutrophils could function as a defense mechanism, interacting with the plasma membrane to generate extracellular traps in response to bacterial infections (44). Thus, it is possible that histone proteins in the glucagon interactome take a role in the fusion step of granule exocytosis. Additionally, secretion of histones and other nuclear proteins has been associated with an inflammatory or senescent secretory phenotype (45, 46).

The alpha cell paracrine effectors, GABA and insulin, remodeled the glucagon interactome in α-TC1-6 cells in a manner that was dependent on glucose levels. Compared to the respective control groups, GABA altered >70 and >80% of the metabolic-regulatory-secretory proteins within the glucagon interactome in the context of 25 and 5.5 mM glucose, respectively. One potentially novel GABA-regulated protein that may function in glucagon secretion in 25 mM glucose is ERC1, which has a role in the formation of the cytomatrix active zone and insulin exocytosis from beta cells (47), and we show for the first time a potential inhibitory effect of ERC1 on glucagon secretion that may be dependent on GABA. Ohara-Imaizumi et al. showed that ERC1 depletion in MIN6 cells and rat pancreatic β-cells suppressed glucose stimulated insulin secretion (47). When pancreatic β-cells were exposed to high glucose conditions, ERC1 takes a role in the process of granule docking and fusion toward insulin exocytosis. Here, by showing that depletion of ERC1 increased glucagon secretion at 25 mM glucose and reduced it at 5.5 mM glucose, it is tempting to speculate that ERC1 is a part of the granule exocytosis machinery in alpha cells and plays a potential role in controlling glucagon exocytosis under diabetic conditions. Another potentially novel player in GABA-regulated glucagon secretion is KCIP-1, associated with beta cell survival (48). Furthermore, our proteomics findings suggest that GABA may enhance glucose uptake and glucose tolerance through leucine-rich repeat proteins. These proteins bind to the insulin receptor to promote glucose uptake in beta cells (49), and thus may be a new paracrine, or even autocrine, regulator of alpha cell function. Interestingly, in the context of 5.5 mM glucose, GABA recruited PCSK2 and secretogranin 2, known alpha cell granule proteins that function in proglucagon processing (19). Although our previous work showed no changes in PCSK2 in response to 5.5 mM glucose (17), we now show that plasticity in PCSK2 expression may be due to GABA under these glucose concentrations.

In the context of 25 mM glucose, insulin treatment increased the number of biosynthetic proteins, consistent with its role in cellular growth. Kinesin-like proteins also increased, suggesting a potential role in alpha cell secretory granule synthesis and glucose homeostasis, as has been documented in beta cells (50). In the context of 5.5 mM glucose, insulin up-regulated nucleoside diphosphate kinases A and B, proposed regulators of insulin secretion (51). We also identified the small G proteins SAR1, Rab2A, and RhoA, present in INS-1 cell secretory granules (52); however, their functions are not known.

Interestingly, treatment of the α-TC1-6 cells with GABA + insulin in 25 mM glucose caused a dramatic decrease in the overall numbers of proteins within the glucagon interactome. Interaction with GRP78 remained preserved, while a new protein, microtubule-associated protein 2, appeared in the glucagon interactome. This protein may have a potential role in glucose homeostasis, as it is down-regulated in isolated diabetic rat islets exposed to low glucose conditions (53). In the context of 5.5 mM glucose, the combination of GABA and insulin again predicted the presence of PCSK2 in the glucagon interactome, as seen with GABA treatment alone and invites revisiting the question of PCSK2 acting as a sorting receptor for glucagon (19).

The design of our experiments was to mimic blood glucose volatility in diabetes in particular, and not in normal physiology, to investigate potential dynamic alterations in the glucagon interactome (54). Here, we have identified the glucagon interactome in α-TC1-6 cells after chronic exposure to extremely high glucose (25 mM), which, in diabetes, paradoxically increases glucagon secretion from pancreatic alpha cells (3, 5). We further showed remodeling of this interactome by replacing that extremely high glucose condition (25 mM) with a relatively low glucose (5.5 mM) medium, which mimic conditions that represent glucose volatility in diabetes. However, we did not examine changes in the glucagon interactome throughout a range of high and low glucose conditions, which could be a limitation for the current study. Also, we used our negative control, Fc alone, only in the two glucose conditions and not in treatments with GABA and insulin, which may affect the interpretation of the interactome under these conditions.

It is well-established that, under normal physiological conditions, glucagon secretion is suppressed by high glucose (21). However, chronic hyperglycemia disrupts this fine regulation and results in elevated glucagon secretion (34, 55). It has been documented that chronic exposure to 25 mM glucose stimulates glucagon secretion in α-TC1-6 cells (17), thus mimicking the alpha cell response to glucose in the diabetic, and not normal, condition.

While we presented a novel glucagon interactome within enriched secretory granules of α-TC1-6 cells and its alterations due to nutritional or paracrine effectors, direct comparisons to primary alpha cells may be limited. When we compared our described glucagon interactome with the transcriptomic profile of mouse alpha cells (56), and human α-cells (57), there were some differences in the protein profiles. Additionally, Lawlor et al. (58) compared gene expression profiles of α-TC1 cells with their primary mouse and human counterparts and showed a high level of discrepancy between them. One possibility for this discrepancy may be changes in gene expression in primary cells while cultured *in vitro* (17, 59). However, we feel that the findings we are reporting generally show that: (1) networks of proteins can interact with glucagon within the secretory granule compartment of the pancreatic α-cell; (2) this interactome is remodeled according to the micro-environmental milieu; and (3) some proteins within the interactome can regulate glucagon secretion. The next step will be to use our data to guide the identification of glucagon-interacting proteins that may regulate glucagon secretion within primary alpha cells.

Under normal physiological conditions, GABA and insulin suppress glucagon secretion in pancreatic α-cells (21, 60). This response to GABA and insulin may differ depending on the cell line and experimental conditions used. Piro et al. (61) showed that with short-term treatment, insulin significantly suppressed glucagon secretion in α-TC1-6 cells without affecting cellular glucagon content. In INR1G cells, Kawamori et al. (62) showed that silencing of the insulin receptor significantly increased glucagon secretion, indicating that insulin receptor signaling is required for suppression of glucagon secretion. Here, we show that treatment with insulin suppresses the long-term cumulative secretion of glucagon when α-TC1-6 cells were cultured and chronically kept in 25 mM glucose. Interestingly, under these conditions, cellular glucagon content increased, perhaps due to excess glucagon in the medium and its potential abolishing effect on insulin action (63). In addition, this increase could be due to an autocrine effect of glucagon on proglucagon gene expression, a notion that has been argued by Leibiger et al. for short-term effect of glucagon on proglucagon gene expression in non-cumulative culturing (26). As well, it is known that GABA inhibits glucagon secretion under high glucose conditions (64). Importantly, our findings show reductions in both glucagon secretion and content. Surprisingly, the combination of GABA and insulin did not suppress glucagon secretion, leading to questions on the mechanism of the interactions between these two signaling pathways.

In conclusion, we have described a novel and dynamic glucagon interactome in α-TC1-6 cells that is remodeled in response to glucose and the alpha cell paracrine effectors, GABA and insulin. Our proteomics approach has revealed a number of novel secretory granule proteins that function in the regulation of glucagon secretion and illustrates the plasticity in the protein components of the alpha cell secretory granules. These findings provide an important proteomics resource for further data mining of the alpha cell secretory granules and targeting diabetes treatment.

## Author Contributions

FA and SD designed the experiments, wrote, and prepared the manuscript text and figures, and reviewed the manuscript prior to submission.

### Conflict of Interest Statement

The authors declare that the research was conducted in the absence of any commercial or financial relationships that could be construed as a potential conflict of interest.
