# Supplementary material for: Plasticity in the Glucagon Interactome Reveals Novel Proteins That Regulate Glucagon Secretion in α-TC1-6 Cells
Source: Front Endocrinol (Lausanne). 2019 Jan 18;9:792. doi: 10.3389/fendo.2018.00792 (PMC6346685; doi:10.3389/fendo.2018.00792)
Supplement: Supplementary file 1 [file Table_1.pdf]

Supplementary Table 1: Reagents and resources.

REAGENT or RESOURCE	SOURCE	IDENTIFIER	Research Resource Identification number
GABA	Sigma	Cat # A2129	
Insulin	Sigma	Cat # 105-16	
ApogeeMix beads	Apogee FlowSystems Inc.	Cat # 1493	
Lipofectamine 2000	Invitrogen	Cat # 11668-027	
Acetonitrile	BDH	Cat # 83639.100E	
Formic acid	Optima	Cat # A117-50	
Dithiotreitol	Fisher Scientific	Cat # BP172-5	
Iodoacetamide	Alfa Aesar	Cat # A14715	
Protein A Sepharose CL-4B	GE Healthcare Life Science	Cat # 71-7090-00 AF	
HYPERSEP C18 column	ThermoFisher Scientific	Cat # 60108-303	
Mini Protease Inhibitor Cocktail	Sigma-Aldrich	Cat # 11836153001	
Trypsin	Promega	Cat # V5111	

Supplementary Table 1 (Continued)

**REAGENT or RESOURCE**	**SOURCE**	**IDENTIFIER**	**Research Resource Identification number**
Collagen type 1	Sigma	Cat # C3867	
NuPAGE	Invitrogen	Cat # NP0335Box	
iBlot Gel Transfer stacks, PVDF	Invitrogen	Cat # IB401001	
Anti-GRP78 Bip (HSPA5) antibody	Abcam	Cat # Ab21685	
Anti-VAMP2 antibody	Abcam	Cat # ab181869	AB_ 2721005
Anti-TGN46 antibody	Abcam	Cat # 1605	AB_443307
Anti-LaminB1 antibody	Abcam	Cat # ab16048	AB_ 443298
Anti-Histone H4 antibody (ChIP grade)	Abcam	Cat # ab10158	AB_ 296888
Anti-Glucagon antibody	Abcam	Cat # Ab10988	AB_ 297642
ProLong Gold antifade reagent with DAPI	Molecular Probes	Cat # P36935	

Supplementary Table 1 (Continued)

REAGENT or RESOURCE	SOURCE	IDENTIFIER	Research Resource Identification number
AlexaFluor 488 goat anti-mouse IgG	Molecular Probes	Cat # A-11001	AB_ 2534069
AlexaFluor 488 goat anti-rabbit IgG	Molecular Probes	Cat # A-11012	AB_ 2534079
Fc-specific FITC antibody	Sigma	Cat # F4143	AB_ 259587
Anti-ERC1 antibody	Abcam	Cat # ab 180507	
Anti- MDH1 antibody	Abcam	Cat # ab 180152	
Anti-ATP5A antibody	Abcam	Cat # ab 176569	
Anti-Aconitase 2 antibody	Abcam	Cat # ab 129105	
Anti-peroxiredoxin2 antibody	Abcam	Cat # ab 109367	
Anti-TUBA1B antibody	Abcam	Cat # ab 108629	
Anti-14-3-3zeta antibody	Abcam	Cat # ab 51129	

Supplementary Table 1 (Continued)

**REAGENT or RESOURCE**	**SOURCE**	**IDENTIFIER**	**Research Resource Identification number**
Anti Na/K ATPase antiody	Thermo Fisher Scientific	Cat# PA5-75640	
Anti-PDI antibody	Abcam	Cat # ab 3672	
Anti-Calreticulin antibody	Abcam	Cat # ab 2907	
Anti-Histone H4 antibody	Abcam	Cat # ab 10158	
**Software and Algorithms**			
Panther Classification System		http://Pantherdb.org	SCR_015893
ExPASY Bioinformatics Resource Portal		http://string-db.org	SCR_015894
UniportKB		http://uniport.org	SCR_004426
STRING		https://string-db.org	SCR_005223
**Experimental model: cell line**			
Alpha TC1-6 cell		mouse pancreatic α-cell line	

Supplementary Table 1 (Continued)

REAGENT or RESOURCE	SOURCE	IDENTIFIER	Research Resource Identification number
**Recombinant DNA**			
FC_pcDNA3.1(+)		http://www.genscript.com	
FC-glucagon_pcDNA3.1(+)		http://www.genscript.com	
**Commercial assays**			
Glucagon assay kit	ThermoFisher Scientific	Cat # EHGCG	
BCA protein assay kit	Biovision Incorporated	Cat # K813-2500	
Histone H4 Modification Multiplex Assay Kit	Abcam	Cat # Ab185914	
RNeasy extraction kit	Qiagen	Cat # 74104	
SuperScript III First Strand Synthesis Supermix	Thermo Fisher Scientific	Cat # 11752050	
Maxima SYBR Green qPCR Master Mix	Thermo Fisher Scientific	Cat # K0221	
Maxima SYBR Green qPCR Master Mix	Thermo Fisher Scientific	Cat # K0221	

siRNA			
14-3-3 zeta/delta	Thermo Fisher Scientific	Cat # S76190<br>Cat # S76191<br>Cat # S76189	
Aconitase	Thermo Fisher Scientific	Cat # S61847<br>Cat # S61845<br>Cat # S61846	

Supplementary Table 1 (Continued)

REAGENT or RESOURCE	SOURCE	IDENTIFIER	Research Resource Identification number
Sodium-Potassium transporting subunit gamma	Thermo Fisher Scientific	Cat # S62726<br>Cat # S201066<br>Cat # S62725	
Protein disulfide-isomerase	Thermo Fisher Scientific	Cat # S71205<br>Cat # S71206	
Peroxiredoxin-2	Thermo Fisher Scientific	Cat # S204749<br>Cat # S232273<br>Cat # S232272	
Malate dehydrogenase	Thermo Fisher Scientific	Cat # S69980	

cytoplasmic		Cat # S69981<br>Cat # S69979	
Aconitate hydratase mitochondrial	Thermo Fisher Scientific	Cat # S61847<br>Cat # S61845<br>Cat # S61846	
ELKS/Rab6-interacting/CAST family member 1	Thermo Fisher Scientific	Cat # S200972<br>Cat # S99972<br>Cat # S99973	

Supplementary Table 1 (Continued)

**REAGENT or RESOURCE**	**SOURCE**	**IDENTIFIER**	**Research Resource Identification number**
Tubulin alpha-1B chain	Thermo Fisher Scientific	Cat # S202331<br>Cat # S202330<br>Cat # S75582	
ATP synthase subunit alpha mitochondrial	Thermo Fisher Scientific	Cat # S62742<br>Cat # S62744<br>Cat # S62743	
Histone H4	Thermo Fisher Scientific	Cat # S234377<br>Cat # S234480<br>Cat # S234061	

GCG (PPG)	Thermo Fisher Scientific	Cat# s66523<br>Cat# s66522<br>Cat# s66524	
β-Actin	Thermo Fisher Scientific	Cat# s61899<br>Cat# s200989<br>Cat# s200988	
Mission siRNA Universal Negative Control #1	Sigma	SIC001	
