# Supplementary material for: Plasticity in the Glucagon Interactome Reveals Novel Proteins That Regulate Glucagon Secretion in α-TC1-6 Cells
Source: Front Endocrinol (Lausanne). 2019 Jan 18;9:792. doi: 10.3389/fendo.2018.00792 (PMC6346685; doi:10.3389/fendo.2018.00792)
Supplement: Supplementary file 2 [file Table_2.pdf]

Supplementary Table 2: List of proteins that interact the with Fc segment alone under conditions of 25 mM glucose.

Associated proteins with Fc segment in 25 mM glucose containing medium
Endoplasmin, Protein disulfide-isomerase A6, Leucine zipper protein 1, Eukaryotic initiation factor 4A-II, DnaJ homolog subfamily C member 12, UPF0565 protein C2orf69 homolog, Obg-like ATPase 1, Catenin beta-1, Nascent polypeptide-associated complex subunit alpha, muscle-specific form, Protein scribble homolog, AP2-associated protein kinase 1, Drebrin-like protein, Sorting nexin-2, Nucleoporin NUP53, Transient receptor potential cation channel subfamily M member 6, Transcription elongation factor A protein-like 3, Tyrosine-protein phosphatase non-receptor type 23, Protein phosphatase 1G, T-complex protein 1 subunit zeta, Malate dehydrogenase, mitochondrial, Protein CutA, Transaldolase, Dihydropyrimidinase-related protein 2, 60S ribosomal protein L10-like, Probable cation-transporting ATPase 13A1, 40S ribosomal protein S30, Phosphatidylethanolamine-binding protein 1, RNA-binding protein 14, Synaptosomal-associated protein 25, 60S ribosomal protein L8, Phosphatidylinositol-binding clathrin assembly protein, Importin subunit beta-1, T-complex protein 1 subunit delta, Proliferating cell nuclear antigen, Zyxin, Non-histone chromosomal protein HMG-17, Aldose reductase, T-complex protein 1 subunit eta, Sulfated glycoprotein 1, Stress-induced-phosphoprotein 1, Peroxiredoxin-1, 40S ribosomal protein S6, Ataxin-2-like protein, C-1-tetrahydrofolate synthase, cytoplasmic, Endophilin-B1, ProSAAS, Histidine triad nucleotide-binding protein 1, Transitional endoplasmic reticulum ATPase, Desmoplakin, Myosin-10, Clathrin light chain A, 26S protease regulatory subunit 6A, Rab GDP dissociation inhibitor alpha, Eukaryotic translation initiation factor 4 gamma 1, N-acetylglucosamine-6-sulfatase, Histone H3.3C, Eukaryotic initiation factor 4A-I, Transketolase, 60S ribosomal protein L22, Glucosidase 2 subunit beta, Dolichyl-diphosphooligosaccharide--protein glycosyltransferase 48 kDa subunit, Nuclear transport factor 2, Golgin subfamily A member 4, Tryptophan--tRNA ligase, cytoplasmic, Eukaryotic translation initiation factor 4B, Tripeptidyl-peptidase 2, Ubinuclein-2, V-type proton ATPase subunit G 1, Peroxiredoxin-4, Aminoacyl tRNA synthase complex-interacting multifunctional protein 1, Potassium voltage-gated channel subfamily C member 3, Afadin, Nuclear autoantigenic sperm protein, 60S ribosomal protein L19, Histone H3.1, Vacuolar protein sorting-associated protein VTA1 homolog, Coatomer subunit alpha, COP9 signalosome complex subunit 3, Thioredoxin domain-containing protein 17, Serine/threonine-protein kinase PAK 2, Rho-associated protein kinase 2, G protein-regulated inducer of neurite outgrowth 1, Pleckstrin homology domain-containing family G member 5, F-actin-capping protein subunit beta, Histone H1.3, Microtubule-associated protein 4, Septin-9, Stromal membrane-associated protein 2, Trifunctional purine biosynthetic protein adenosine-3, Eukaryotic initiation factor 4A-III, Creatine kinase B-type, Chromatin target of PRMT1 protein,

Supplementary Table 2 (continued)

Associated proteins with Fc segment in 25 mM glucose containing medium
Calreticulin, Phosphoglycerate mutase 1, 40S ribosomal protein S17, Eukaryotic translation initiation factor 3 subunit B, E3 ubiquitin-protein ligase HUWE1, Valine--tRNA ligase, Basigin, ADP-ribosylation factor GTPase-activating protein 2, Melanoma inhibitory activity protein 3, Profilin-1, DNA replication licensing factor MCM4, Heterogeneous nuclear ribonucleoprotein M, Protein disulfide-isomerase A4, Protein YIPF5, Emerin, Multifunctional protein ADE2, Tubulin-specific chaperone A, Protein transport protein Sec31A, 60S ribosomal protein L10, Secretory carrier-associated membrane protein 3, GTP-binding nuclear protein Ran, Phosphoglycerate kinase 2, Ribosome-binding protein 1, Leucine-rich repeat-containing protein 40, Nucleoside diphosphate kinase B, T-complex protein 1 subunit epsilon, Carboxypeptidase E, Phosphoglycolate phosphatase, Regulator complex protein LAMTOR1, 60S ribosomal protein L24, 40S ribosomal protein S27-like, Poly(rC)-binding protein 2, V-type proton ATPase catalytic subunit A, Myosin light polypeptide 6, Ubiquitin-associated protein 2, Protein DJ-1, Beta-synuclein, 60S ribosomal protein L7, Ubiquitin-like modifier-activating enzyme 1, 60S ribosomal protein L3, V-type proton ATPase 116 kDa subunit a isoform 1, Palmitoyltransferase ZDHHC5, General vesicular transport factor p115, WD and tetratricopeptide repeats protein 1, NADH-cytochrome b5 reductase 3, Nestin, Poly(rC)-binding protein 3, Rho GDP-dissociation inhibitor 1, Serine/arginine repetitive matrix protein 2, Guanine nucleotide-binding protein-like 1, Replication protein A 14 kDa subunit, Exportin-2, Cytoplasmic dynein 1 intermediate chain 2, Fragile X mental retardation protein 1 homolog, Unconventional myosin-Ia, Vigilin, Cofilin-1, Fatty acid synthase, 40S ribosomal protein S5, UMP-CMP kinase, 40S ribosomal protein S27, Putative RNA-binding protein Luc7-like 2, Actin-related protein 3, Neutral alpha-glucosidase AB, Plasminogen activator inhibitor 1 RNA-binding protein, 40S ribosomal protein SA, Matrin-3, Elongation factor 2, NudC domain-containing protein 3, 60S ribosomal protein L14, Neurofilament medium polypeptide, Protein LYRIC, Nucleolin, 40S ribosomal protein S12, 60S ribosomal protein L4, Protein FAM135B, Histone H1.4, Protein transport protein Sec61 subunit beta, Vacuolar protein sorting-associated protein 35, Latent-transforming growth factor beta-binding protein 3, Moesin, Insulin receptor substrate 2, 15 kDa selenoprotein, T-complex protein 1 subunit gamma, UPF0160 protein MYG1, mitochondrial, Microtubule-associated tumor suppressor candidate 2 homolog, Hsp90 co-chaperone Cdc37, Proteasome subunit beta type-7, Heat shock protein 105 kDa, Tetraspanin-13, 60S ribosomal protein L12, Low molecular weight phosphotyrosine protein phosphatase, Constitutive coactivator of PPAR-gamma-like protein 1, Synaptic vesicle membrane protein VAT-1 homolog, Heterogeneous nuclear ribonucleoprotein A/B, Toll-interacting protein, Pyruvate dehydrogenase E1 component subunit beta, mitochondrial, Gamma-enolase, LDLR chaperone MESD, Guanine nucleotide-binding protein G(s) subunit alpha isoforms XLas, Alanine--tRNA ligase, cytoplasmic, Vesicle-fusing ATPase, Tumor protein D52, Rab GDP dissociation inhibitor beta, NEDD8-conjugating enzyme Ubc12, Cathepsin D, Epidermal growth factor receptor substrate 15-like 1, Xaa-Pro aminopeptidase 1, Splicing factor 1, Copine-1, Thioredoxin domain-containing protein 5, Microtubule-associated protein 1B, 6-phosphogluconolactonase, Vesicle-associated membrane protein 2, Glycylpeptide N-tetradecanoyltransferase 1, Peptidyl-prolyl cis-trans isomerase FKBP8, ATP synthase subunit d, mitochondrial, Neuroendocrine convertase 2, Protein ELFN1, 60S ribosomal protein L6, Growth factor receptor-bound protein 10, Serine/threonine-protein phosphatase 2A 65 kDa regulatory subunit A alpha isoform, Vesicle-associated membrane protein 4,

Supplementary Table 2 (Continued)

Associated proteins with Fc segment in 25 mM glucose containing medium
Microtubule-associated protein RP/EB family member 1, 60S ribosomal protein L7a, Talin-1, Alpha-actinin-4, Aminoacyl tRNA synthase complex-interacting multifunctional protein 2, Syntaxin-12, Splicing factor, proline- and glutamine-rich, Bifunctional purine biosynthesis protein PURH, Homeobox protein engrailed-2, Nuclease-sensitive element-binding protein 1, Glutamine synthetase, Ras-related protein Rab-7a, 14-3-3 protein theta, Sjoegren syndrome/scleroderma autoantigen 1 homolog, Mitochondrial import receptor subunit TOM34, Heterogeneous nuclear ribonucleoprotein U, 10 kDa heat shock protein, mitochondrial, Septin-2, Annexin A5, Calcyclin-binding protein, Bromodomain-containing protein 4, Serine/arginine-rich splicing factor 4, Glyoxylate reductase/hydroxypyruvate reductase, Ras-related protein Rab-1A, Chromobox protein homolog 3, Dolichyl-diphosphooligosaccharide--protein glycosyltransferase subunit 1, Attractin, Glucose-6-phosphate isomerase, Protein capicua homolog, DNA replication licensing factor MCM6, Ubiquitin carboxyl-terminal hydrolase 5, Prefoldin subunit 2, Uncharacterized protein C20orf201 homolog, Histone-lysine N-methyltransferase 2D, Adenylyl cyclase-associated protein 1, Thioredoxin domain-containing protein 12, Ras-related protein Rab-5C, 60S ribosomal protein L18, 60S ribosomal protein L29, 40S ribosomal protein S11, Proteasomal ubiquitin receptor ADRM1, Cyclin-dependent kinase 12, Clathrin heavy chain 1, Leucine-rich repeat-containing protein 59, Prohibitin, Secretogranin-2, Vesicle-associated membrane protein-associated protein B, Ferritin light chain 1, 60S ribosomal protein L26, Microtubule-actin cross-linking factor 1, CD2 antigen cytoplasmic tail-binding protein 2, Spectrin beta chain, non-erythrocytic 1, Transmembrane emp24 domain-containing protein 4, Transmembrane emp24 domain-containing protein 9, Cytochrome b-c1 complex subunit 2, mitochondrial, Myristoylated alanine-rich C-kinase substrate, Vesicle-associated membrane protein 3, 60S ribosomal protein L21, Leucine-rich repeat-containing protein 47, Transcription elongation factor A protein-like 5, Delta and Notch-like epidermal growth factor-related receptor, PRA1 family protein 2, ATP-dependent RNA helicase DDX3X, Histone H3.3, Calnexin, Nucleosome assembly protein 1-like 4, 60S ribosomal protein L5, Protein canopy homolog 2, ATP-citrate synthase, Histone H3.2, Clathrin light chain B, Serine/arginine-rich splicing factor 5, Glyceraldehyde-3-phosphate dehydrogenase. Neurofilament light polypeptide, Adenosylhomocysteinase, Guanine nucleotide-binding protein G(o) subunit alpha, Fructose-bisphosphate aldolase A, Poly(rC)-binding protein 1, COMM domain-containing protein 3, Cathepsin Z, , Cleavage and polyadenylation specificity factor subunit 6, V-type proton ATPase subunit E 1, Zinc finger SWIM domain-containing protein 8, Serine--tRNA ligase, cytoplasmic, Acetyl-CoA acetyltransferase, mitochondrial, Alpha-taxilin, Ras-related protein Rap-1b, Nuclear pore complex protein Nup155
