# Supplementary material for: Plasticity in the Glucagon Interactome Reveals Novel Proteins That Regulate Glucagon Secretion in α-TC1-6 Cells
Source: Front Endocrinol (Lausanne). 2019 Jan 18;9:792. doi: 10.3389/fendo.2018.00792 (PMC6346685; doi:10.3389/fendo.2018.00792)
Supplement: Supplementary file 3 [file Table_3.pdf]

Supplementary Table 3: List of proteins that interact the with Fc segment alone under conditions of 5.5 mM glucose.

Succinyl-CoA:3-ketoacid coenzyme A transferase 1, mitochondrial, Histone-lysine N-methyltransferase SETD1B, Importin subunit beta-1, Aldose reductase, Tumor-associated calcium signal transducer 2, Heterogeneous nuclear ribonucleoprotein A3, Elongation factor 1-gamma, 60S ribosomal protein L22, Calmodulin, Ubiquitin-conjugating enzyme E2 N, Actin, alpha skeletal muscle, Peptidyl-prolyl cis-trans isomerase FKBP2, V-type proton ATPase catalytic subunit A, Ubiquitin-like modifier-activating enzyme 1, Nascent polypeptide-associated complex subunit alpha, L-lactate dehydrogenase B chain, Farnesyl pyrophosphate synthase, Protein FAM171A2, Msx2-interacting protein, T-complex protein 1 subunit gamma, Calreticulin, Electron transfer flavoprotein subunit alpha, mitochondrial, ER membrane protein complex subunit 2, 40S ribosomal protein S2, L-lactate dehydrogenase A chain, Acyl-CoA-binding protein, Serine/threonine-protein phosphatase 2A 65 kDa regulatory subunit A alpha isoform, L-lactate dehydrogenase C chain, Alpha-actinin-4, Nuclease-sensitive element-binding protein 1, Chromogranin-A, Prefoldin subunit 2, Protein FAM117B, Stromal interaction molecule 2, ATP-dependent RNA helicase DDX3X, Nucleosome assembly protein 1-like 4, 60S ribosomal protein L5, Protein SET, Neurofilament light polypeptide, Transgelin-2
