# Supplementary material for: Plasticity in the Glucagon Interactome Reveals Novel Proteins That Regulate Glucagon Secretion in α-TC1-6 Cells
Source: Front Endocrinol (Lausanne). 2019 Jan 18;9:792. doi: 10.3389/fendo.2018.00792 (PMC6346685; doi:10.3389/fendo.2018.00792)
Supplement: Supplementary file 4 [file Table_4.pdf]

Supplementary Table 4: Profile of the histone, cytoskeletal and ribosomal proteins contained within the glucagon interactome when αTC1-6 cells were incubated for 24h in media containing 25 mM or 5.5 mM glucose.

25 mM glucose containing medium	5.5 mM glucose containing medium
Histone H2A type 1, Histone H2A type 1-F, Histone H2A type 1-F, Histone H2A type 1-H, Histone H2A type 1-K, Histone H2A type 2-A, Histone H2A type 2-B, Histone H2A type 2-C, Histone H2A type 3, Histone H2A.J, Histone H2AX, Histone H2B type 1-B, Histone H2B type 1-C/E/G, Histone H2B type 1-F/J/L, Histone H2B type 1-H, Histone H2B type 1-K, Histone H2B type 1-M, Histone H2B type 1-P, Histone H2B type 2-B, Histone H2B type 2-E, Histone H2B type 3-A, Histone H2B type 3-B<br><br>Actin cytoplasmic 2, Actin cytoplasmic 1, Actin alpha skeletal muscle, Actin aortic smooth muscle, Actin alpha cardiac muscle 1, Actin gamma-enteric smooth muscle, Beta-actin-like protein 2, Tubulin beta-4B chain, Tubulin alpha-1C chain, Tubulin alpha-1A chain, Tubulin alpha-1B chain, Tubulin beta-5 chain, Tubulin beta-2B chain, Tubulin beta-4A chain, Tubulin beta-3 chain, Tubulin beta-2A chain<br><br>60S ribosomal protein L13, 40S ribosomal protein S14, 60S ribosomal protein L23a, 60S ribosomal protein L11, 60S acidic ribosomal protein P1, Elongation factor 1-delta, Elongation factor 1-alpha 2, Eukaryotic translation initiation factor 5A-1, Elongation factor 1-alpha 1	Histone H2A type 3, Histone H2A type 1-F, Histone H2B type 1-B, Histone H1.3, Histone H2A type 2-A, Histone H2B type 1-H, Histone H2B type 3-A, Histone H4, Histone H3.3, Histone H3.3C, Histone H2A.J, Histone H2A type 1-K, Histone H3.1, Histone H2A type 2-C, Histone H2A type 1, Histone H2B type 1-K, Histone H3.2, Histone H2B type 1-M, Histone H1.2, Histone H2B type 3-B, Histone H2B type 2-B, Histone H2B type 2-E, Histone H2B type 1-C/E/G, Histone H2A type 1-H, Histone H1.5, Histone H2B type 1-P, Histone H1.1, Histone H2A type 2-B, Histone H2A type 1-F, Histone H2B type 1-B, Histone H2AX, Histone H2B type 1-F/J/L<br><br>Actin, cytoplasmic 1, Actin, cytoplasmic 2, Tubulin beta-4B chain, Tubulin beta-4A chain, Tubulin beta-5 chain, Tubulin alpha-1C chain, Tubulin beta-3 chain, Tubulin alpha-1A chain, Tubulin alpha-1B chain<br><br>40S ribosomal protein S15a, 60S ribosomal protein L23a, 40S ribosomal protein S7, 40S ribosomal protein S25, 60S ribosomal protein L13, 40S ribosomal protein S14, 40S ribosomal protein S12, Elongation factor 1-alpha 2, 60S acidic ribosomal protein P1, 60S ribosomal protein L11, 60S ribosomal protein L18, 60S acidic ribosomal protein P2, Elongation factor 2, Ubiquitin-60S ribosomal protein L40, Eukaryotic translation initiation factor 4B
