# Supplementary material for: Plasticity in the Glucagon Interactome Reveals Novel Proteins That Regulate Glucagon Secretion in α-TC1-6 Cells
Source: Front Endocrinol (Lausanne). 2019 Jan 18;9:792. doi: 10.3389/fendo.2018.00792 (PMC6346685; doi:10.3389/fendo.2018.00792)
Supplement: Supplementary file 5 [file Table_5.pdf]

Supplementary Table 5: Profile of the histone, cytoskeletal and ribosomal proteins within the glucagon interactome. αTC1-6 cells were cultured in media containing 25 mM glucose and treated with GABA (Table S4-A), insulin (Table S4-B) and GABA+ insulin (Table S4-C). Proteins were identified using LC-MS/MS.

Supplementary Table 5-A

GABA + 25 mM glucose
Histone H2A type 1, Histone H2A type 1-F, Histone H2A type 1-F, Histone H2A type 1-H, Histone H2A type 1-K, Histone H2A type 2-A, Histone H2A type 2-B, Histone H2A type 2-C, Histone H2A type 3, Histone H2A.J, Histone H2AX, Histone H2B type 1-B, Histone H2B type 1-C/E/G, Histone H2B type 1-F/J/L, Histone H2B type 1-H, Histone H2B type 1-K, Histone H2B type 1-M, Histone H2B type 1-P, Histone H2B type 2-B, Histone H2B type 2-E, Histone H2B type 3-A, Histone H2B type 3-B, Histone H4<br><br>Actin cytoplasmic 1, Actin cytoplasmic 2, Anionic trypsin-2, Tubulin alpha-1A chain, Tubulin alpha-1B chain, Tubulin alpha-1C chain, Tubulin beta-2A chain, Tubulin beta-2B chain, Tubulin beta-3 chain, Tubulin beta-4A chain, Tubulin beta-4B chain, Tubulin beta-5 chain<br><br>60S acidic ribosomal protein P1, 60S ribosomal protein L11, Elongation factor 1-alpha 1, Eukaryotic translation initiation factor 4E

Supplementary Table 5-B

Insulin + 25 mM glucose
Histone H1.5, Histone H1t, Histone H2A type 1, Histone H2A type 1-F, Histone H2A type 1-F, Histone H2A type 1-H, Histone H2A type 1-K, Histone H2A type 2-A, Histone H2A type 2-B, Histone H2A type 2-C, Histone H2A type 3, Histone H2A.J, Histone H2AX, Histone H2B type 1-B, Histone H2B type 1-C/E/G, Histone H2B type 1-F/J/L, Histone H2B type 1-H, Histone H2B type 1-K, Histone H2B type 1-M, Histone H2B type 1-P, Histone H2B type 2-B, Histone H2B type 2-E, Histone H2B type 3-A, Histone H2B type 3-B, Histone H4<br><br>Actin alpha cardiac muscle 1, Actin alpha skeletal muscle, Actin aortic smooth muscle, Actin cytoplasmic 1, Actin cytoplasmic 2, Actin gamma-enteric smooth muscle, Tubulin alpha-1A chain, Tubulin alpha-1B chain, Tubulin alpha-1C chain, Tubulin beta-2A chain, Tubulin beta-2B chain, Tubulin beta-3 chain, Tubulin beta-4A chain, Tubulin beta-4B chain, Tubulin beta-5 chain<br><br>40S ribosomal protein S14, 60 kDa heat shock protein mitochondrial, 60S acidic ribosomal protein P1, 60S ribosomal protein L11, Elongation factor 1-alpha 1, Elongation factor 1-alpha 2, Eukaryotic translation initiation factor 5A-1

Supplementary Table 5-C

GABA+ insulin + 25 mM glucose
Histone H2B type 1-B, Histone H2B type 1-B, Histone H2B type 1-C/E/G, Histone H2B type 1-F/J/L, Histone H2B type 1-H, Histone H2B type 1-K, Histone H2B type 1-M, Histone H2B type 1-P, Histone H2B type 2-B, Histone H2B type 2-B, Histone H2B type 2-E, Histone H2B type 3-A, Histone H2B type 3-B<br><br>Actin alpha cardiac muscle 1, Actin alpha skeletal muscle, Actin aortic smooth muscle, Actin cytoplasmic 1, Actin cytoplasmic 1, Actin gamma-enteric smooth muscle, Tubulin alpha-1A chain, Tubulin alpha-1A chain, Tubulin alpha-1B chain, Tubulin alpha-1C chain, Tubulin alpha-1C chain<br><br>60S ribosomal protein L23a
