# Supplementary material for: Plasticity in the Glucagon Interactome Reveals Novel Proteins That Regulate Glucagon Secretion in α-TC1-6 Cells
Source: Front Endocrinol (Lausanne). 2019 Jan 18;9:792. doi: 10.3389/fendo.2018.00792 (PMC6346685; doi:10.3389/fendo.2018.00792)
Supplement: Supplementary file 6 [file Table_6.pdf]

Supplementary Table 6: Profile of the histone, cytoskeletal and ribosomal proteins within the glucagon interactome. αTC1-6 cells were cultured in media containing 5.5 mM glucose and treated with GABA (Table S5-A), insulin (Table S5-B) and GABA+insulin (Table S5-C). Proteins were identified using LC-MS/MS.

Supplementary Table 6-A

GABA + 5.5 mM glucose
Histone H1.3, Histone H1.5, Histone H2A type 1, Histone H2A type 1-F, Histone H2A type 1-H, Histone H2A type 1-K, Histone H2A type 2-A, Histone H2A type 2-C, Histone H2A type 3, Histone H2A.J, Histone H2B type 1-B, Histone H2B type 1-C/E/G, Histone H2B type 1-F/J/L, Histone H2B type 1-H, Histone H2B type 1-K, Histone H2B type 1-P, Histone H2B type 2-B, Histone H2B type 2-E, Histone H2B type 3-A, Histone H2B type 3-B, Histone H3.1, Histone H3.3, Histone H3.3C, Histone H4, Histone H1.2, Histone H2B type 1-B, Histone H2B type 1-M, Histone H3.2<br>Actin cytoplasmic 1, Actin cytoplasmic 2, Tubulin alpha-1A chain, Tubulin alpha-1B chain, Tubulin alpha-1C chain, Tubulin beta-3 chain, Tubulin beta-5 chain<br>40S ribosomal protein S14, 40S ribosomal protein S15, 40S ribosomal protein S25, 40S ribosomal protein S5, 40S ribosomal protein SA, 60S acidic ribosomal protein P1, 60S acidic ribosomal protein P2, 60S ribosomal protein L11, 60S ribosomal protein L15, 60S ribosomal protein L18, 60S ribosomal protein L7a, 60S ribosomal protein L7a, Ubiquitin-60S ribosomal protein L40, Eukaryotic initiation factor 4A-I, Eukaryotic initiation factor 4A-II, Eukaryotic initiation factor 4A-III, Elongation factor 1-alpha 1, Elongation factor 2

Supplementary Table 6-B

Insulin + 5.5 mM glucose
Histone H1.1, Histone H1.2, Histone H1.3, Histone H1.4, Histone H1.5, Histone H1t, Histone H2A type 1, Histone H2A type 1-F, Histone H2A type 1-H, Histone H2A type 1-K, Histone H2A type 2-A, Histone H2A type 2-C, Histone H2A type 3, Histone H2A.J, Histone H2B type 1-B, Histone H2B type 1-C/E/G, Histone H2B type 1-F/J/L, Histone H2B type 1-H, Histone H2B type 1-K, Histone H2B type 1-M, Histone H2B type 1-P, Histone H2B type 2-B, Histone H2B type 3-A, Histone H2B type 3-B, Histone H3.1, Histone H3.2, Histone H3.3, Histone H3.3C, Histone H4<br>Actin alpha cardiac muscle 1, Actin alpha skeletal muscle, Actin aortic smooth muscle, Actin cytoplasmic 1, Actin cytoplasmic 2, Actin gamma-enteric smooth muscle, Tubulin alpha-1A chain, Tubulin alpha-1B chain, Tubulin alpha-3 chain, Tubulin alpha-4A chain, Tubulin beta-5 chain, Tubulin-specific chaperone A<br>40S ribosomal protein S12, 40S ribosomal protein S14, 40S ribosomal protein S15a, 40S ribosomal protein S18, 40S ribosomal protein S19, 40S ribosomal protein S20, 40S ribosomal protein S25, 40S ribosomal protein S3, 40S ribosomal protein S3, 40S ribosomal protein S3a, 40S ribosomal protein S8, 40S ribosomal protein SA, 60 kDa heat shock protein mitochondrial, 60S acidic ribosomal protein P1, 60S acidic ribosomal protein P2, 60S ribosomal protein L11, 60S ribosomal protein L12, 60S ribosomal protein L13, 60S ribosomal protein L15, 60S ribosomal protein L18, 60S ribosomal protein L23a, 60S ribosomal protein L26, 60S ribosomal protein L27, 60S ribosomal protein L7, 60S ribosomal protein L7, 60S ribosomal protein L9, Elongation factor 1-alpha 1, Elongation factor 1-alpha 2, Elongation factor 1-beta, Elongation factor 1-delta, Elongation factor 2, Eukaryotic initiation factor 4A-I, Eukaryotic initiation factor 4A-II, Eukaryotic initiation factor 4A-III, Transcription elongation factor A protein-like 3, Transcription elongation factor A protein-like 3, Transcription elongation factor A protein-like 5, Transcription factor SOX-1,

Supplementary Table 6-C

GABA+ insulin + 5.5 mM glucose
Histone H1.5, Histone H2A type 1, Histone H2A type 1-F, Histone H2A type 1-H, Histone H2A type 1-K, Histone H2A type 2-A, Histone H2A type 2-C, Histone H2A type 3, Histone H2A.J, Histone H2AX, Histone H2B type 1-B, Histone H2B type 1-C/E/G, Histone H2B type 1-H, Histone H2B type 1-M, Histone H2B type 1-P, Histone H2B type 2-B, Histone H2B type 3-A, Histone H2B type 3-B, Histone H4, Histone H2A type 2-B, Histone H2B type 1-F/J/L<br>Actin cytoplasmic 1, Tubulin alpha-1A chain, Tubulin alpha-1B chain, Tubulin alpha-1C chain, Tubulin beta-2A chain, Tubulin beta-2B chain, Tubulin beta-3 chain, Tubulin beta-4A chain, Tubulin beta-4B chain, Tubulin beta-5 chain<br>60S ribosomal protein L11, Elongation factor 1-alpha 1, Elongation factor 2
