# Supplementary material for: Plasticity in the Glucagon Interactome Reveals Novel Proteins That Regulate Glucagon Secretion in α-TC1-6 Cells
Source: Front Endocrinol (Lausanne). 2019 Jan 18;9:792. doi: 10.3389/fendo.2018.00792 (PMC6346685; doi:10.3389/fendo.2018.00792)
Supplement: Supplementary file 7 [file Table_7.pdf]

Supplementary Table 7: Functional categories of the proteins within the glucagon interactome in the context of 25 mM glucose. Proteins were functionally categorized using Panther GO-Slim Molecular Function analysis. Values show protein hits as percentage of the total number of hits within each category when αTC1-6 cells were cultured in media containing 25 mM glucose.

	Control	GABA	Insulin	GABA+ insulin
Binding	47.4	46.1	48.5	51.4
Structural molecule activity	24.7	13.7	15.4	28.6
Catalytic activity	19.6	22.5	26.2	17.1
Receptor activity	1	5.9	2.3	-
Translation regulator activity	4.1	1	2.3	-
Transporter activity	3.1	6.9	4.6	2.9
Signal transducer activity	-	2.9	-	-
Antioxidant activity	-	1	0.8	-
