# Supplementary material for: Plasticity in the Glucagon Interactome Reveals Novel Proteins That Regulate Glucagon Secretion in α-TC1-6 Cells
Source: Front Endocrinol (Lausanne). 2019 Jan 18;9:792. doi: 10.3389/fendo.2018.00792 (PMC6346685; doi:10.3389/fendo.2018.00792)
Supplement: Supplementary file 8 [file Table_8.pdf]

Supplementary Table 8: Functional categories of proteins within the glucagon interactome in the context of 5.5 mM glucose. Proteins were functionally categorized using Panther GO-Slim Molecular Function analysis. Each value shows protein hit as percentage of the total number of hits within each category when αTC1-6 cells were cultured in media containing 5.5 mM glucose.

	Control	GABA	Insulin	GABA+ insulin
Binding	48.4	43.8	43.5	50.6
Structural molecule activity	15	11.4	14.9	17.6
Catalytic activity	25.5	28.6	31	22.4
Receptor activity	2	1.6	1.2	1.2
Translation regulator activity	2.6	1.1	2	2.4
Transporter activity	3.9	4.9	4.3	4.7
Antioxidant activity	2	1.6	1.6	1.2
Channel regulator activity	-	0.5	-	-
Signal transducer activity	-	6.5	1.6	-
