# Supplementary material for: Plasticity in the Glucagon Interactome Reveals Novel Proteins That Regulate Glucagon Secretion in α-TC1-6 Cells
Source: Front Endocrinol (Lausanne). 2019 Jan 18;9:792. doi: 10.3389/fendo.2018.00792 (PMC6346685; doi:10.3389/fendo.2018.00792)
Supplement: Supplementary file 9 [file Presentation_1.pdf]

Supplementary Figure legends

Supplementary Figure 1: Immunoblotting assessment of enrichment in the extracted secretory granules. Western blot analyses of the cell extract **(CE)**, extract of the secretory granules in media containing 25 mM glucose **(E-25)** and 5.5 mM glucose **(E-5.5)**. Proteins were resolved on 4-12% SDS-PAGE gel (NuPAGE), transferred to PVDF membrane and probed with antibodies to identify **(A)** vesicle-associated membrane protein 2 (VAMP2) for mature secretory granules; **(B)** Calreticulin for the endoplasmic reticulum; **(C)** TGN46 for the trans-Golgi network; and **(D)** Lamin B1 for the nuclear envelope. The immunoreactive bands were visualized using HRP-conjugated goat anti-rabbit secondary antibody and Clarity Western ECL substrate.

Supplementary Figure 2: Nano-scale flow cytometry of the enriched secretory granules. **(A)** Calibrating nano flow cytometer for size distribution of the enriched secretory granules using ApogeeMix beads. Circles indicate bead sizes of 110, 179, 235, 304, 585 and 880 nm. **(B)** Gating the glucagon^{+} secretory granules (L488) using FITC secondary antibody against the Fc segment of Fc-glucagon. **(C)** Size distribution of the secretory granules within the gate containing glucagon^{+} secretory granules.

Supplementary Figure 3: siRNA mediated gene silencing of target proteins in the secretory granules. αTC1-6 cells were transfected with 50 nM pooled of 3 predesigned siRNAs. Cell extract subjected to Western blot to follow expression of the following target proteins. For each protein, band intensity of the expressed protein was determined using ImageJ software. Values of ≥ 70% reduction in protein expression levels were considered as successful protein depletion. Alpha-tubulin 2 (AT2), ATP synthase F1 subunit alpha (ATP5F1A), cytosolic malate dehydrogenase (MDH1), Protein disulfide-isomerase (PDI), ELKS/Rab6-interacting/CAST family member 1 (ERC1), Aconitate hydratase mitochondrial (ACO2), peroxiredoxin-2 (PRDX2), FXYD domain-containing ion transport regulator 2 (FXYD2), 14-3-3 zeta/delta (KCIP-1), Histone H4 and Glucose regulated protein 78 KDa (GRP78).

Supplementary Figure 4: Proglucagon gene knock down in αTC1-6 cells. cDNA synthesis was performed using total extracted RNA. Quantitative PCR was achieved using Quant Studio Design and Analysis Real-Time PCR Detection System in conjunction with the Maxima SYBR Green qPCR Master Mix. The glucagon gene (GCG, PPG) expression was determined in the transfected cells with scrambled siRNA (negative control) or GCG siRNA. Glucagon gene expression level was normalized to that of the internal control β-Actin. The normalized level of transcripts in protein depleted cells was shown relative to that of the negative control. Gene expression levels show >70% reduction in the GCG depleted group compare to the control.

Supplementary Figure 5: Alterations in glucagon secretion and cell glucagon content of α-TC1-6 cells in response to nutritional and paracrine effectors. α-TC1-6 cells were cultured and kept under high glucose (25 mM) condition for a long-term. These chronically exposed cells to high glucose condition were treated with GABA (25 µM), insulin (100 pM) or GABA (25 µM) + insulin (100 pM) for 24h in 25 mM or 5.5 mM glucose containing media. At the end of incubation, glucagon levels were measured in the media (**5A**) and cell extract (**5B**) using ELISA. Values were expressed as mean ± SD (n=4-5) and compared among groups using one-Way ANOVA (α=0.05).*$p<0.05$, **$p<0.01$. ***$p<0.001$.
