# Supplementary figures and images for: Plasticity in the Glucagon Interactome Reveals Novel Proteins That Regulate Glucagon Secretion in α-TC1-6 Cells
Source: Front Endocrinol (Lausanne). 2019 Jan 18;9:792. doi: 10.3389/fendo.2018.00792 (PMC6346685; doi:10.3389/fendo.2018.00792)

Supplementary Figure 1

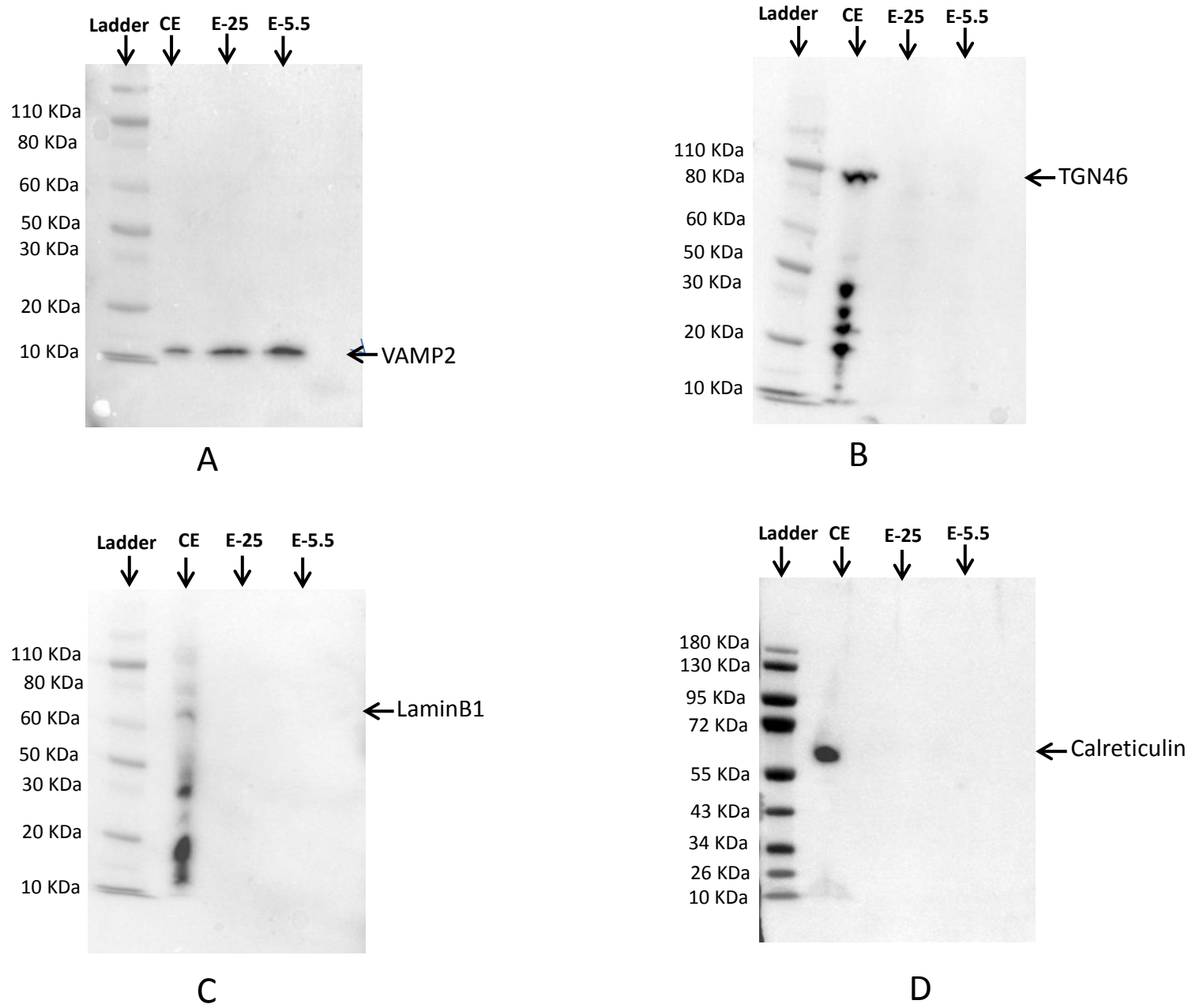

Supplement: Supplementary file 10 [file Image_1.pdf]

Supplementary Figure 2

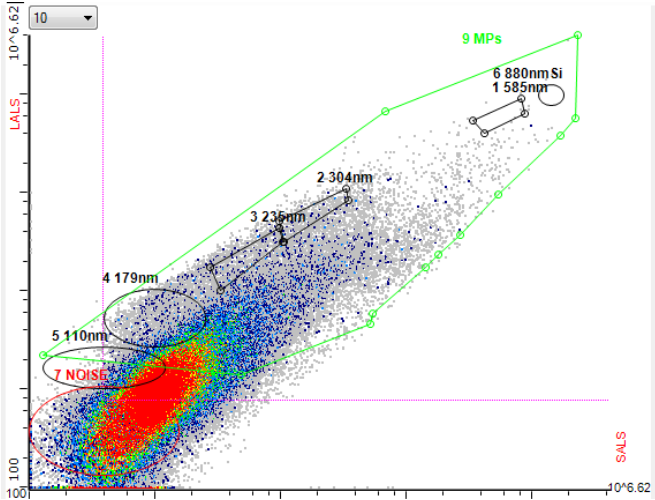

A

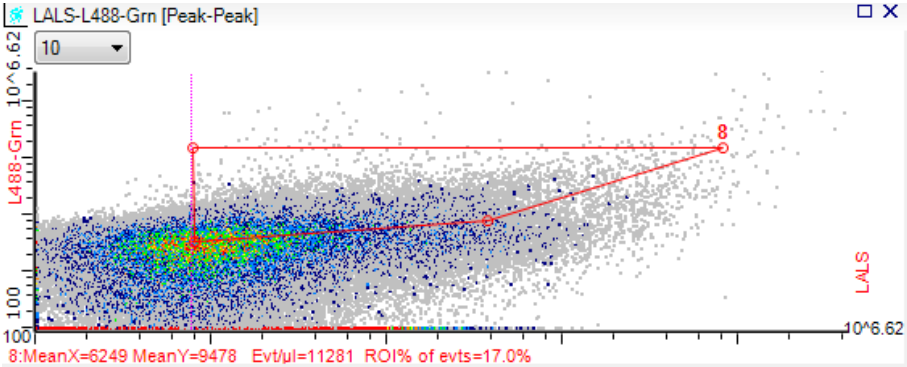

B

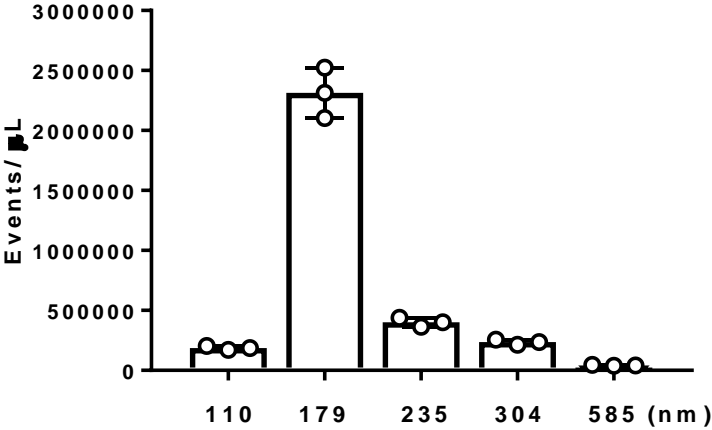

C

Supplement: Supplementary file 11 [file Image_2.pdf]

Supplementary Figure 3

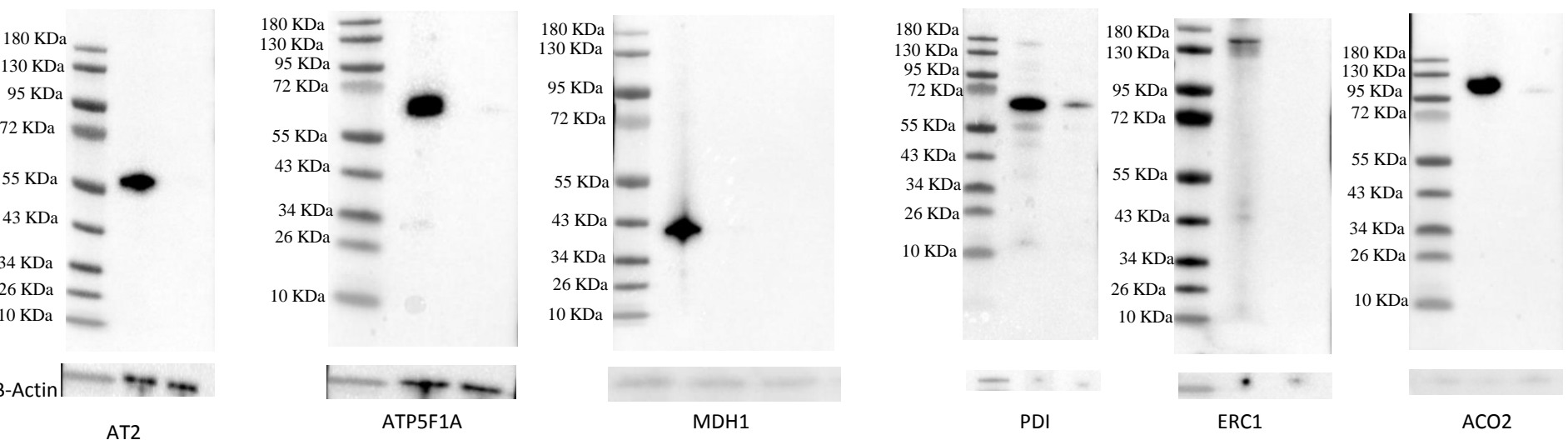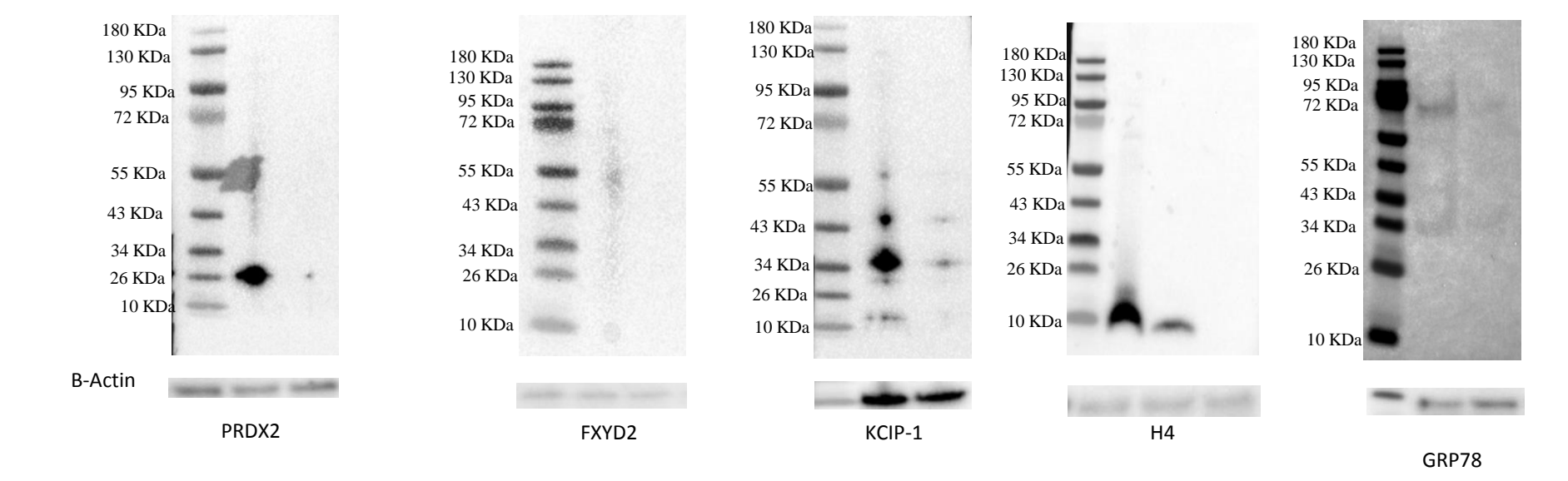

Supplement: Supplementary file 12 [file Image_3.pdf]

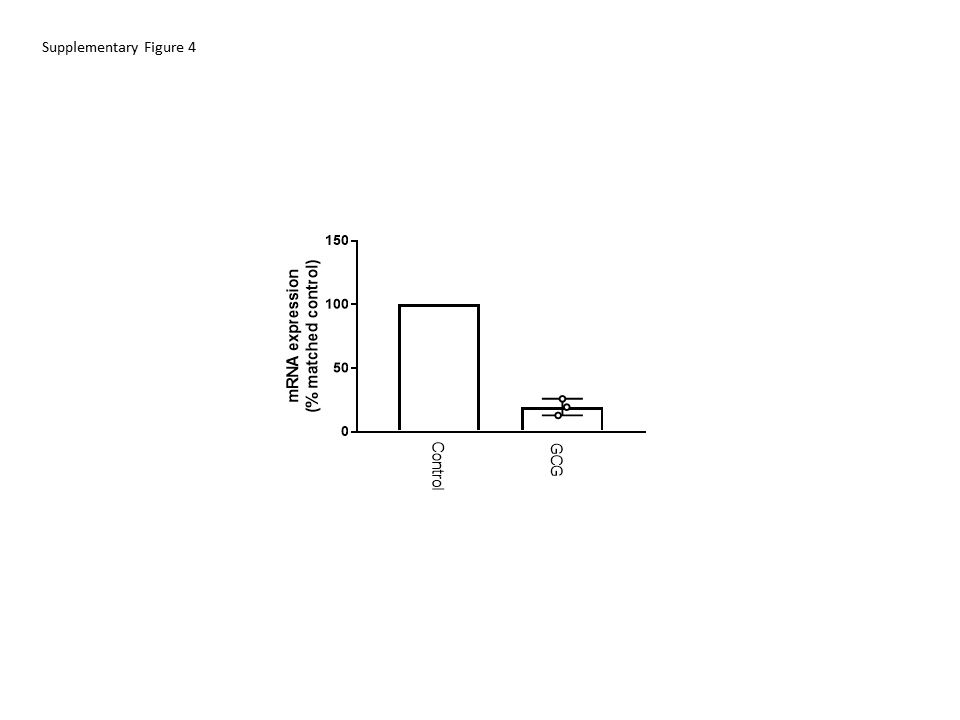

Supplement: Supplementary file 13 [file Image_4.tif]

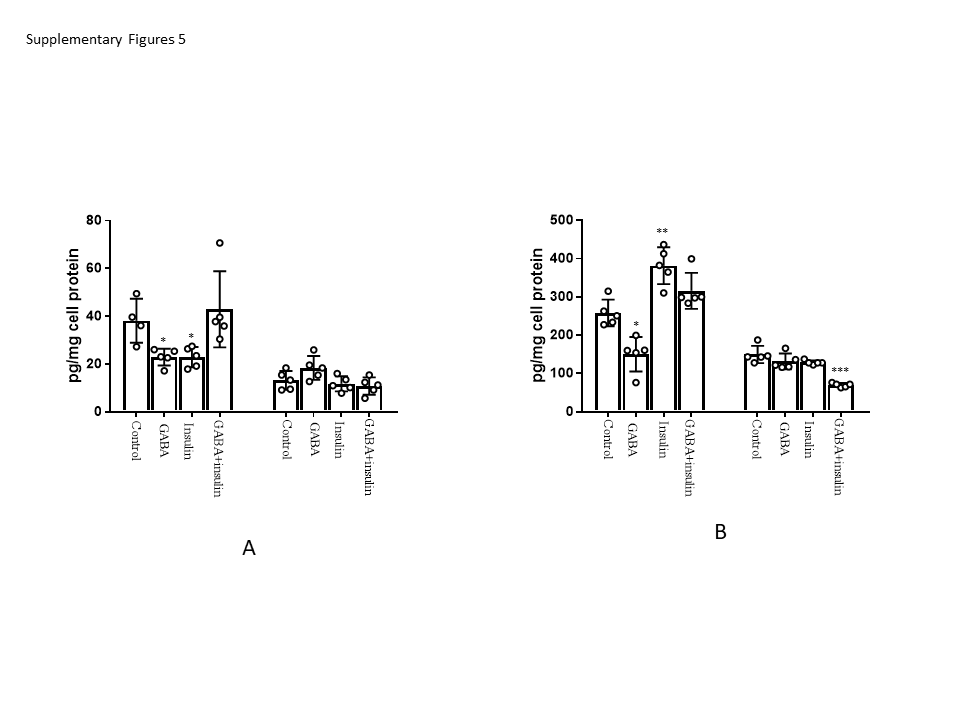

Supplement: Supplementary file 14 [file Image_5.tif]
